# Tenascin C as a novel zinc finger protein 750 target regulating the immunogenicity via DNA damage in lung squamous cell carcinoma

**DOI:** 10.1186/s12885-024-12285-8

**Published:** 2024-05-06

**Authors:** Lu Xia, Hexin Lin, Huifen Cao, Jiabian Lian

**Affiliations:** 1 Xiamen Cell Therapy Research Center, The First Affiliated Hospital of Xiamen University, School of Medicine, Xiamen University, Xiamen, 361000 CN China; 2 Department of Gastrointestinal Oncology Surgery, The First Affiliated Hospital of Xiamen University, School of Medicine, Xiamen University, Xiamen, 361000 CN China; 3 Department of Colorectal Surgery, The First Affiliated Hospital of Fujian Medical University, Fuzhou, 350000 CN China; 4 Institute of Genomics, School of Medicine, Huaqiao University, Xiamen, 361000 CN China; 5 Department of Clinical Laboratory, The First Affiliated Hospital of Xiamen University, School of Medicine, Xiamen University, Xiamen, 361000 CN China

**Keywords:** DNA damage repair, Hippo/ERK signaling pathway, Immunogenicity of cancer cells, Lung squamous cell carcinoma (LUSC), Tenascin C (TNC), Zinc finger protein 750 (ZNF750)

## Abstract

Modulation of DNA damage repair in lung squamous cell carcinoma (LUSC) can result in the generation of neoantigens and heightened immunogenicity. Therefore, understanding DNA damage repair mechanisms holds significant clinical relevance for identifying targets for immunotherapy and devising therapeutic strategies. Our research has unveiled that the tumor suppressor zinc finger protein 750 (ZNF750) in LUSC binds to the promoter region of tenascin C (TNC), leading to reduced TNC expression. This modulation may impact the malignant behavior of tumor cells and is associated with patient prognosis. Additionally, single-cell RNA sequencing (scRNA-seq) of LUSC tissues has demonstrated an inverse correlation between ZNF750/TNC expression levels and immunogenicity. Manipulation of the ZNF750-TNC axis in vitro within LUSC cells has shown differential sensitivity to CD8^+^ cells, underscoring its pivotal role in regulating cellular immunogenicity. Further transcriptome sequencing analysis, DNA damage repair assay, and single-strand break analyses have revealed the involvement of the ZNF750-TNC axis in determining the preference for homologous recombination (HR) repair or non-homologous end joining (NHEJ) repair of DNA damage. with involvement of the Hippo/ERK signaling pathway. In summary, this study sheds light on the ZNF750-TNC axis's role in DNA damage repair regulation in LUSC, laying a groundwork for future translational research in immune cell therapy for LUSC.

**Supplementary Information:**

The online version contains supplementary material available at 10.1186/s12885-024-12285-8.

## Introduction

Zinc finger protein 750 (ZNF750) regulates epidermal differentiation through the p63-ZNF750-KLF4/KDM1A axis under normal physiological conditions, impacting stem cell differentiation and skin integrity [1, 2]. Notably, ZNF750 has been validated as a lineage-specific tumor suppressor in squamous cell carcinoma [3]. Extensive research has focused on ZNF750 mutations across different cohorts and its mechanism in head and neck squamous cell carcinoma or esophageal squamous cell carcinoma [4–9]. However, limited investigation has been conducted on ZNF750 expression in LUSC, which is crucial for a comprehensive understanding of its tumorigenesis.

Tenascin C (TNC) is an extracellular matrix glycoprotein characterized by a highmolecular weight. It forms disulfide-linked hexameric strucutres and exhibits elevated expression level within the tumor microenvironment, correlating with poor patient survival outcomes across various cancer types [10]. In mouse models, augmentation of Tnc levels via CRISPR-mediated transcriptional activation of endogenous genes has been shown to enhance the metastasis of lung adenocarcinoma cells [11]. Moreover, research has indicated that heightened TNC expression promotes lung metastases in osteosarcoma [12] and breast tumors [12, 13]. Collectively, these findings underscore the pivotal regulatory role of TNC in lung cancer development and lung metastasis establishment.

In this study, we conducted a comprehensive analysis of transcriptome sequencing and chromatin co-immunoprecipitation sequencing (ChIP-seq) of LUSC cells with ZNF750 overexpression. Our investigation revealed that ZNF750 transcriptionally represses the expression of TNC. Our findings demonstrate that ZNF750 and TNC modulate tumor proliferation, invasion, metastasis, and immunogenicity. Clinically, the expression of ZNF750-TNC axis bears prognostic significance. Furthermore, our analysis unveiled that the ZNF750-TNC axis influenced DNA damage repair and further correlated with the Hippo/ERK signaling pathway. Notably, in LUSC cell lines overexpressing ZNF750 or TNC, the DNA break signal in the ZNF750 genomic binding region was significantly diminished compared to the control group.

## Materials and methods

### Cell lines and drugs

The following is a list of the cell lines used in the study: NCI-H226, SK-MES-1, NCI-H1650, NCI-H1975, HBE, LC713 and 293T. These cells were purchased from the National Collection of Authenticated Cell Cultures (Shanghai, China). They were tested and authenticated by Genetic Testing Biotechnology (Suzhoujianda, Suzhou, China). The H226, 1650, and 1975 cells were cultured according to the supplier’s recommendations in RPMI 1640 medium (#C3010-0500, VivaCell) supplemented with 10% fetal bovine serum (FBS). The HBE, SK-MES-1, and 293T cells were cultured according to the supplier’s recommendations in DMEM (#C11995500, Gibco, USA) supplemented with 10% FBS. Additionally, for 293T cells, 1×MEM NEAA (#11140-050, Gibco) was added. LC713 cells were derived from a patient’s pleural effusion and cultured in RPMI 1640 medium supplemented with 10% FBS. Establishing patient-derived cell lines LC713 involves centrifuging the pleural effusion from a patient with lung adenocarcinoma to collect cell sedimentation, followed by the removal of red blood cells and cell debris. The collected cells are then cultured in cell line medium (consisting of DMEM/F12 (Gibco) medium supplemented with 2 mM Glutamax™, 25 mM HEPES (4-(2-hydroxyethyl)-1-piperazineethanesulfonic acid), 10% heat-inactivated FBS (Fetal Bovine Serum) and 33 mM sodium bicarbonate (Gibco)) to promote growth.

Stable cell line(s) expressing ZNF750 (or TNC) were established by infecting lung cancer cells with viral particles containing ZNF750/TNC or empty vector. The cells were then selected with 100 μg/mL Zeocin^®^ or 1 μg/mL puromycin dihydrochloride. TRE-ZNF750 was a tetracycline-inducible expression vector, and the working concentration of Doxycycline Hyclate was 200ng/mL. Vertiporfin was used at a concentration of 1μM, and ERKi was used at a concentration of 5μM. The bioactive molecules used are listed in Supplimentary Table 1.

### ChIP-seq and ChIP-qPCR

In 5 × 10^7^ H226 cells, 1% formaldehyde was used for 10 minutes at room temperature for ZNF750 ChIP. Fixation was neutralized by adding glycine and incubated for 5 minutes at room temperature, followed by washing with phosphate buffered saline twice. Chromatin DNA was sheared to 300-500 bp average in size through sonication for 30 minutes using Bioruptor(5 s on, 5 s off for 125 cycles). The supernatant was immunoprecipitated with anti-ZNF750 antibody overnight at 4 ℃, followed by incubation with protein A/G magnetic beads (#HY-K0202, MedChemExpress) for an additional 2hrs. After washing and elution, the crosslinked protein-DNA complex was reversed by heating at 65 ℃ with shaking overnight (Thermomixer, USA). Immunoprecipitated DNA was purified by using QIAquick PCR Purification Kit (Qiagen, #28104, Netherlands) and subjected to high throughput sequencing or quantitative PCR. The PCR primers used are listed in Supplementary Table 1.

### Single-Strand DNA breaktome profiling

The single-strand DNA breaktome provides a new dimension of biological assessment that can reflect both single-strand breaks and double-strand breaks in DNA (represented by the corresponding sites on the complementary strands). Most (65-77%) single-strand DNA breaks (SSBs) are found in genes or regulatory elements (promoters, enhancers and insulators) covered by ENCODE [14, 15]. For the SSiNGLe library preparation, three million H226 cells were seeded at 1 million cells per ml of medium per well in 6-well plates. After 24 hours, the DNA was extracted directly from these cells using the TIANamp Genomic DNA Kit (TIANGEN Biotech, DP304) according to the manufacturer’s protocol. One hundred nanograms of the DNA were used directly as input and then underwent Illumina library construction following the SSiNGLe-ILM protocol (https://protocolexchange.researchsquare.com/article/pex-920/v2).All SSB profiling experiments were performed on the Illumina NovaSeq platform using paired-end 150 bp strategy at 1GB scale. This was carried out by Novogene Corporation (Beijing).

### Single-Strand DNA breaks mapping

The assignment of SSBs to genomic positions for the data generated in this work was performed using the same method as in the SSiNGLe-ILM protocol, with the exception that an additional filter was applied: only read pairs where the first base of read 2 aligned to the genome were used. All analyses were performed using unique genomic positions of SSBs as previously described [16]. For SSB signal mapping to Exon, TSS, and ZNF750 binding sites, we calculated the number of SSBs or hotspots located within specific types of genomic elements: 1) within ±1000 bp of TSSs; 2) exonic regions; 3) ChIP-seq regions of ZNF750 protein in H226 cells. Exon, TSS were defined based on the longest transcript of each gene from all annotated human genes, irrespective of expression status. The enrichment of overlaps relative to random chance was calculated using the *odds ratio (ORi)*, which was previously detailed [16]. Metascape was applied to conduct the transcriptional factors enrichment analysis of SSBs in ZNF750 binding regions.

### Immune cells killing assay

Peripheral blood was collected from healthy volunteers at the First Affiliated Hospital of Xiamen University. The extraction method of peripheral blood mononuclear cells (PBMC) and the activation and identification of CD8^+^ T cells are detailed in previous studies [17].

Cell index (CI) experiments were performed using the xCELLigence Real-Time Cell Analysis (RTCA) eSight (Agilent Technologies, USA) at 37°C with 5% CO2. The procedures were performed as follows: add 50 μL of cell culture medium to each of the 96 wells of E-Plate VIEW microplates (# 00300601030, Agilent Technologies); leave the plate in the tissue culture hood for 1 hour in eSight at 37°C with 5% CO2. to ensure the culture medium and plate surface achieve temperature equilibrium; add the prepared 50μL cells which contains 5000 tumor cells (the seeding period) and allowed for tumor cell attachment in the following 16 hours, then 50μL activated CD8+ cells were seeded into each wells at a effector: target ratio of 1:1 or 1:5, and then equilibrate for 20 minutes in eSight to detected the impedance baseline at 1 minute intervals. The impedance was recorded at every 15 min’s intervals. All incubations were performed in triplicates at a final volume of 150 µl.

### DNA damage repair assay

DNA damage repair assay was conducted in LUSC cells using co-transfection of pDRGFP (HR repair reporter plasmid) or pimEJ5GFP (NHEJ repair reporter plasmid) with pCBASceI, followed by treatment with DMSO or 1 μM Verteporfin or 5μM FR180204 for 24 hours. The cells were then harvested for flow cytometry assay.

### Xenograft assay

Female BALB/c nude mice (6 weeks old, 18-20 g) were purchased from Shanghai Slack Laboratory Animal Research Center. Mice were maintained at Xiamen University (Xiamen, China). ZNF750-overexpressing H226 cells and control H226 cells (1E7 cells in each group) were injected subcutaneously. Tumor growth and mice weight were measured twice a week until the end of experiment. Tumor volume was estimated according to the following formula: tumor volume (mm^3^)= L ×W^2^/2, where L is the length and W is the width. To euthanize mice humanely, the use of carbon dioxide inhalation method was applied. First, placed the mouse in a container with a tight-fitting lid, and then slowly released carbon dioxide into the container allowing the mouse to inhale it and lose consciousness. This method is fast and painless, ensuring that the mouse does not suffer prolonged distress. All animal experiments were in compliance with ethical guidelines and approved by the Animal Ethics Committee of Xiamen University.

Additional experimental methods are detailed in Supplementary Materials.

## Results

### Overexpression of ZNF750 in LUSC cell lines significantly mitigates the malignant phenotype of tumor cells

**Fig. 1:**
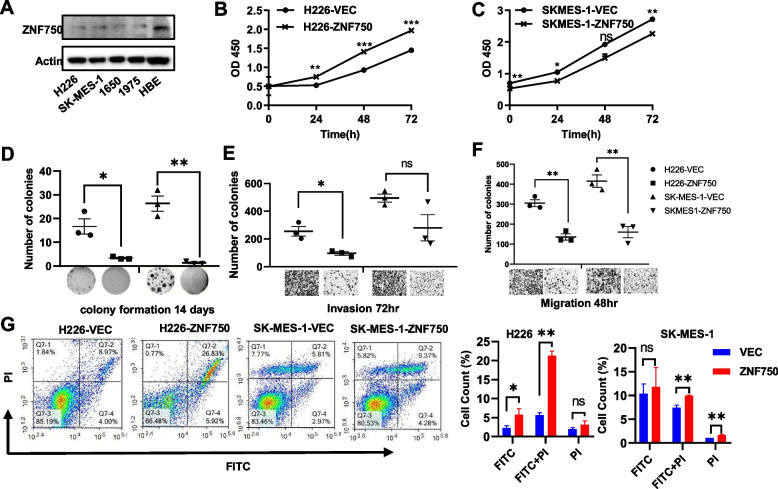
Overexpression of ZNF750 in LUSC cells significantly reduced the malignant phenotype of tumor cells. **A** ZNF750 expression level is higher in normal lung epithelial HBE cell lines than in other lung cancer cell lines. The full-length blots are presented in Supplemental Figures 15-16. **B**-**C** ZNF750 significantly inhibited proliferation, two-way ANOVA. **D** Inhibited colony formation, two sample t test, (**E**-**F**) attenuated the ability of lung squamous carcinoma cells to invade and migrate, two sample t test, (**G**) promoted apoptosis (FITC for early apoptosis, FITC+PI for late apoptosis) of LUSC cells, multiple t test. Significant labels: ns: *P* > 0.05, *: *P*<0.05, **: *P* < 0.01, ***: *P* < 0.001. FITC: Fluorescein Isothiocyanate, PI: Propidium Iodide

*In vitro* studies showed ZNF750 exhibited heightened expression in the normal lung epithelial cell line HBE compared to other lung cancer cell lines (Fig. 1A, Supplemental Figures 15-16), and its expression was comparatively lower in LUSC cells than in LUAD cells (Supplemental Figure 1A). Consistent with expectations, ZNF750 was overexpressed and predominantly localized to the nucleus in LUSC H226 cells (Supplemental Figure 2A). ZNF750 exerted significant inhibitory effects on cell proliferation (Fig. 1B-C), colony formation (Fig. 1D), invasion and migration (Fig. 1E-F), while promoting apoptosis (Fig. 1G), and inducing cell cycle arrest at the G1/S phase (Supplemental Figure 2B-C) in LUSC cells. Furthermore, overexpression of ZNF750 in H226 cells markedly decelerated tumor growth in mice bearing H226 subcutaneous xenograft tumors (Supplemental Figure 2D-F, *P* = 0.003, scale bar = 1 cm). However, phenotypic experiments conducted on LUAD cell lines did not reveal significant differences or even showed contradictory results between ZNF750 overexpressing cells and control cells (Supplemental Figure 2).

**Fig. 2:**
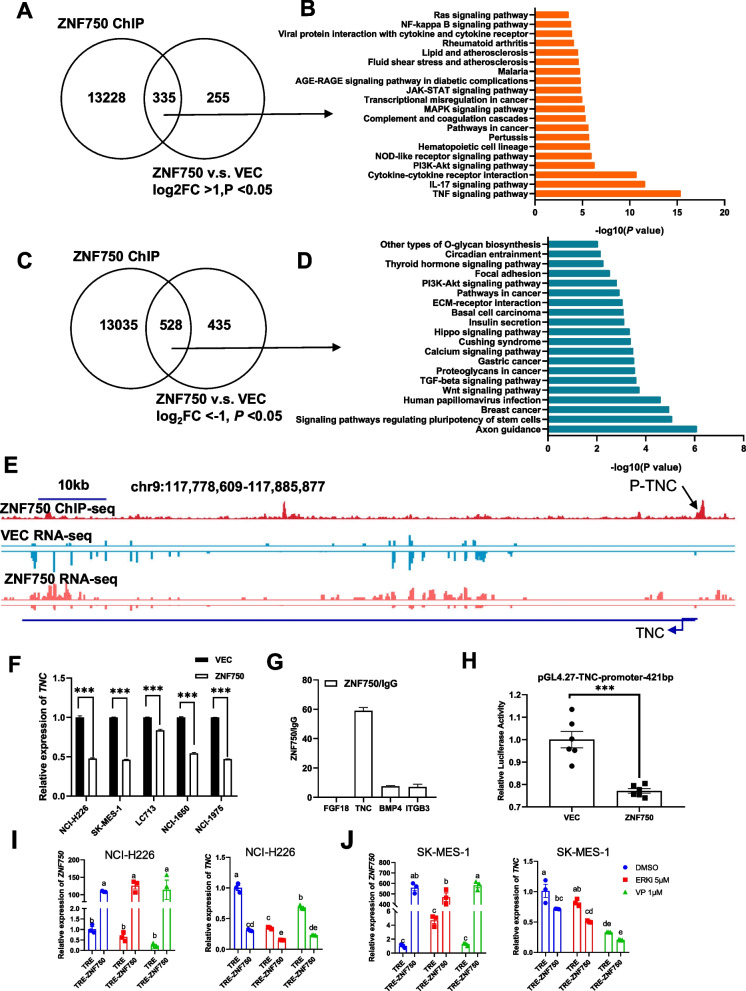
TNC is a direct target gene of ZNF750. **A** Venn diagram showing the overlap between genes from the ChIP-seq analysis of ZNF750 and genes up-regulated by ZNF750 (RNA-seq). **B** KEGG analysis of the top 20 representative pathways up-regulated by ZNF750. **C** Venn diagram showing the overlap between genes from the ChIP-seq analysis of ZNF750 and genes downregulated by ZNF750 (RNA-seq). **D** KEGG analysis of the top 20 representative pathways downregulated by ZNF750. **E** ChIP-seq IGV views of ZNF750 at TNC gene region. The binding sites of primers in (**H**) are indicated as p-TNC in (**E**). **F** ZNF750 overexpression resulted in depression of TNC in 5 indicated lung cancer cell lines, multiple t test. **G** Chromatin immunoprecipitation (ChIP) qPCR assay was performed in H226 cells. **H** Dual luciferase assay of ZNF750 at 421bp fragment of the TNC promoter region, two sample t test. Significant labels: ns: *P* > 0.05, *: *P* <0.05, **: *P* < 0.01, ***: *P* < 0.001. **I**-**J** The ZNF750 and TNC RNA expression transfected with inducible ZNF750 overexpression vector in (**I**) NCI-H226 cells or (**J**)SK-MES-1 cells, two-way ANOVA. Significant labels: Different letter means significant difference between groups, same letter means no significant difference between groups; *P* < 0.05 as significant difference. ERKi: VP: Verteporfin

### ZNF750 binds to transcriptional regulatory regions of TNC and modulates its transcription

Chromatin co-immunoprecipitation (ChIP) experiments conducted in H226 cells yielded high-quality data on ZNF750 binding profiles (Fig. 2E, Supplemental Figure 3). Additionally, we performed transcriptome sequencing (RNA-seq) before and after ZNF750 overexpression to identify candidate genes directly regulated by ZNF750. Moreover, we analyzed the enriched functional pathways of the up-regulated and down-regulated genes following ZNF750 overexpression. The results indicated that 335 up-regulated genes with ZNF750 binding site were predominantly enriched in pathways related to the TNF signaling and cytokine-receptor interaction (Fig. 2A-B). In addition, 528 down-regulated genes with ZNF750 binding sites were mainly enriched in pathways associated with the Wnt signaling, TGF-beta signaling, Hippo signaling, PI3K-Akt signaling, and cell adhesion pathways (Fig. 2C-D).

**Fig. 3:**
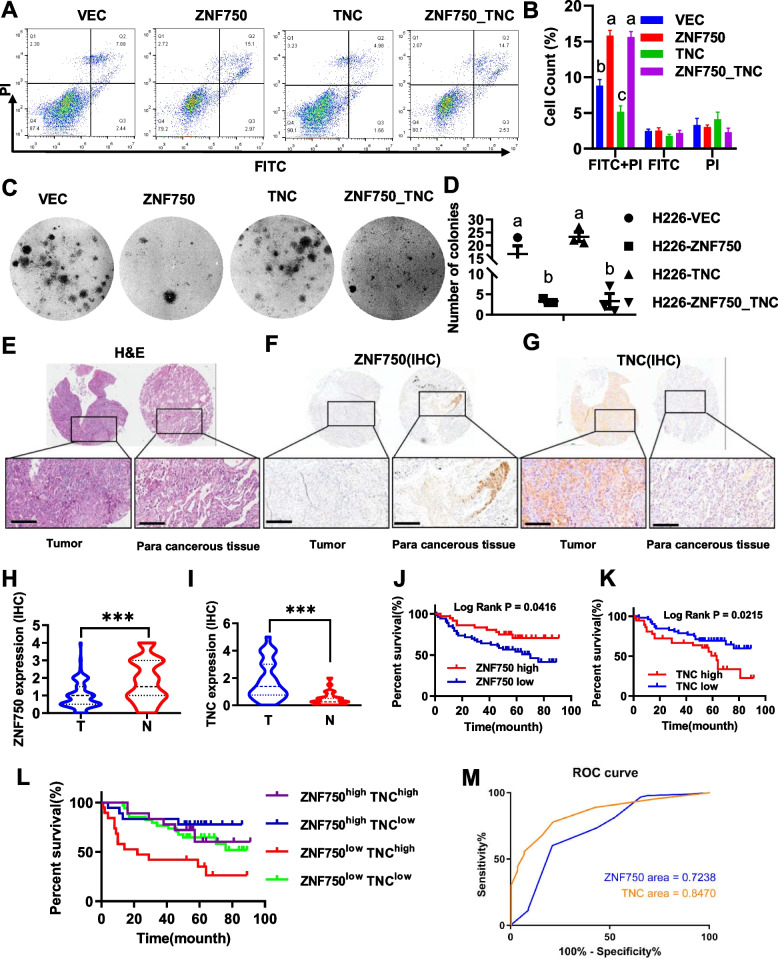
In vitro analysis and the clinical pathologic correlation analysis of ZNF750 and TNC. (**A**-**D**) The effect of ZNF750-TNC axis on the clonogenesis and apoptosis of lung squamous carcinoma cell line, (**A**-**B**) Apoptosis analysis of H226 cells, two-way ANOVA. **C**-**D** the results of clonogenesis of H226 cells showed that TNC could not rescue the reduction of clonogenesis caused by ZNF750, one-way ANOVA. VEC: control group transfected with vehicle vector PCDH, ZNF750: ZNF750 overexpression group transfected with PCDH-ZNF750, TNC: TNC overexpression group, ZNF750_TNC: transfected with both PCDH-ZNF750 and PCDH-TNC. Significant labels: Different letter means significant difference between groups, same letter means no significant difference between groups; *P* < 0.05 as significant difference. (E-M)The clinical pathologic correlation analysis of ZNF750 and TNC. **E**-**G** Representative images of H&E staining (**E**), IHC of ZNF750 (**F**) and IHC of TNC, (*N* = 90, E-G scale bar = 200μm). (**H**-**I**) Quantitative analysis of IHC signal of ZNF750 (**H**) and TNC (**I**), paired t test. **J**-**L** survival analysis based on gene expression level of ZNF750 & TNC, Log-rank test. **M** Diagnostic ROC curve of ZMF750 & TNC

Furthermore, we employed RT-qPCR to validate the expression of genes regulated by ZNF750 in multiple lung cancer cell lines. Genes significantly downregulated by ZNF750 overexpression in at least two out of five tested cell lines (validated by RT-qPCR) are depicted (Fig. 2F, Supplemental Figure 1B, Supplemental Figure 3F-H). Notably, the expression of TNC was markedly downregulated upon ZNF750 overexpression in all five tested cell lines, suggesting the universal regulation of TNC by ZNF750(Fig. 2F). Based on the ChIP-seq binding signals(Fig. 2E, Supplemental Figure 3I-K), further validation through ChIP-qPCR and dual luciferase reporter assays(Fig. 2G-H) confirmed that ZNF750 binds to the TNC promoter and regulates its transcription activity. To investigate the immediate effect of ZNF750 expression on TNC regulation, we utilized a tet-on system to induce ZNF750 expression and observed efficient inhibition of TNC expression in H226 and SK-MES-1 cells within 24 hours of ZNF750 activation(Fig. 2I-J). Additionally, the administration of ERK inhibitor or YAP inhibitor effectively suppressed TNC expression.

### In vitro cellular experiments have elucidated the functional role of the ZNF750-TNC axis, while clinicopathological analysis has identified ZNF750 and TNC as effective prognostic markers.

ZNF750 can counteract the pro-malignant phenotype induced by TNC overexpression (Fig. 3A-D), indicating an inhibitory functional axis of ZNF750-TNC in H226 cells. Consistent with its *in vitro* functional role, clinicopathological analysis of LUSC revealed low expression of ZNF750 in tumor tissue juxtaposed with high expression of TNC(Fig. 3E-I, Supplemental Figure 4). ZNF750 was associated with a favorable prognosis in LUSC, whereas TNC was linked to a poorer prognosis (Fig. 3J-K, Supplemental Figure 5). Notably, when considering ZNF750 and TNC expression jointly, the group exhibiting high ZNF750 expression and low TNC expression displayed the most favorable prognosis, while the group with low ZNF750 expression and high TNC expression exhibited the poorest prognosis(Fig. 3L). Receiver operating characteristic (ROC) curves demonstrated that both ZNF750 and TNC served as effective markers for distinguishing between tumor and paracancerous tissue (Fig 3M). Furthermore, chi-square test revealed a significant correlation between ZNF 750 or TNC expression and tumor differentiation, clinical stages, and invasion depth (Tables 1 & 2). In conclusion, we have affirmed the clinical significance of ZNF750-TNC axis in the development of LUSC.

**Fig. 4:**
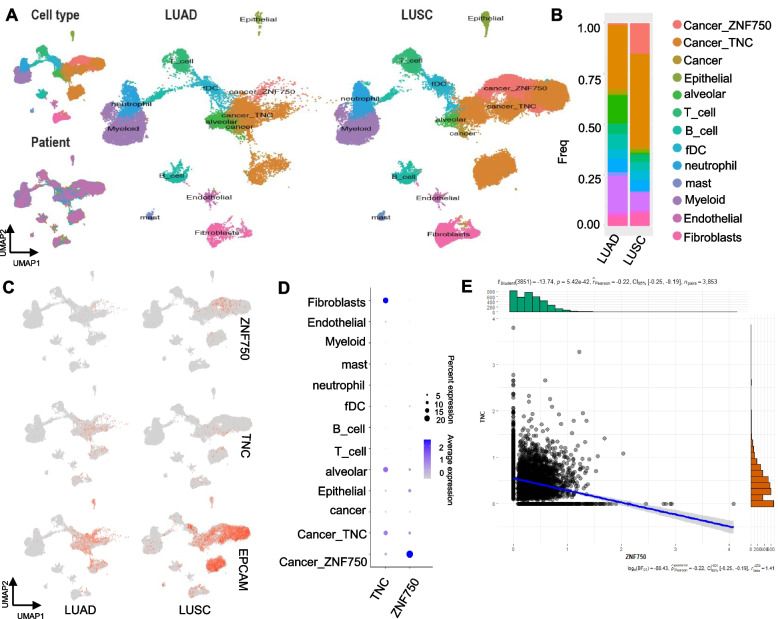
Advanced NSCLC single-cell atlas. **A** UMAP plot of 89887 cells from 20 LUAD and 22 LUSC patients, colored by 13 major cell types. **B** Major cell-type composition of LUAD and LUSC. **C** Feature plots of ZNF750 and TNC of LUAD and LUSC, visualized by UMAP. **D** Dot plot illustrating the average expression level of ZNF750 and TNC across 13 major cell types. **E** Pearson correlation analysis evaluating the association between TNC and ZNF750 expression within cancer cells, conducted using ggstatsplot R package (Pearson's *r* = -0.22)

**Fig. 5:**
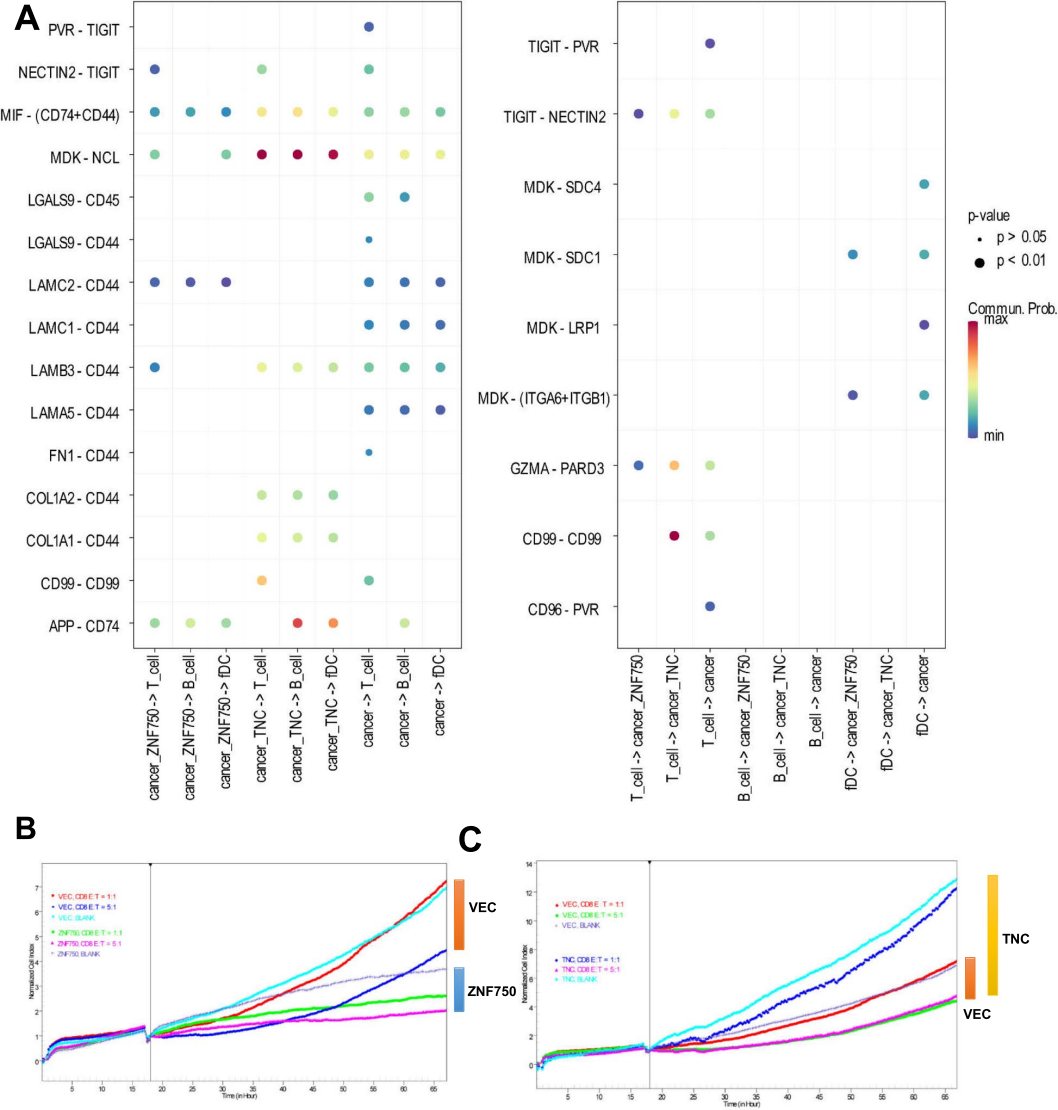
The dynamic interplay within the tumor microenvironment influenced by ZNF750-TNC expression. **A** Molecular communication depicting growth factor interactions between cancer cells and immune cell populations (T cells, B cells, and fDC), analyzed using the CellChat R package (Version 1.5.0). Cellular relationships are represented as "ligand cell -> receptor cell" interactions. **B**-**C** In vitro cytotoxicity assays evaluating the impact of ZNF750 (B) or TNC (C) overexpression on the cytolytic activity of CD8^+^ T cells against lung squamous cell carcinoma cells, in co-culture with peripheral blood mononuclear cells (PBMCs)

**Table 1:** ZNF750 expression and the clinicopathological features of 90 LUSC patients

**Characteristics**	**n**	**ZNF750 expression**		***P*-value**
		**Low *n*=54**	**High *n*=36**	
**Gender**				
Male	84	50	34	0.7301
Female	6	4	2	
**Age(years)**				
<60	29	14	15	0.1175
≥60	61	40	21	
**Tumor differentiation**				
Well	25	7	24	**0.0001**
Moderate or Poor	65	**47**	12	
**Clinical stage**				
I&II	39	**31**	8	**0.0010**
III&IV	51	23	28	
**Invasion depth**				
T1	10	8	2	0.1709
T2-3	80	46	34	
**Lymphovascular invasion**				
Negative	49	32	17	0.2613
Positive	41	22	19	
**Tumor size**				
<5cm	48	32	16	0.1675
≥5cm	42	22	20	
**TNC expression(IHC)**				
Low	53	28	25	0.0966
High	37	26	11	

*P*-valus<0.05 are indicated in bold

**Table 2:** TNC expression and the clinicopathological features of 90 LUSC patients

**Characteristics**	**n**	**TNC expression**		***P*-value**
		**Low *n*=53**	**High *n*=37**	
**Gender**				
Male	84	50	34	0.6469
Female	6	3	3	
**Age(years)**				
<60	29	19	10	0.3782
≥60	61	34	27	
**Tumor differentiation**				
Well	25	8	17	**0.0013**
Moderate or Poor	65	**45**	20	
**Clinical stage**				
I&II	39	24	15	0.6551
III&IV	51	29	22	
**Invasion depth**				
T1	10	10	0	**0.0051**
T2-3	80	**43**	37	
**Lymphovascular invasion**				
Negative	49	31	18	0.6378
Positive	41	22	19	
**Tumor size**				
<5cm	48	29	19	0.8726
≥5cm	42	24	18	
**ZNF750 expression(IHC)**				
Low	54	28	26	0.0966
High	36	25	11	

*P*-valus<0.05 are indicated in bold

### Single-cell transcriptome sequencing (scRNA-seq) has revealed the expression characteristics of ZNF750 and TNC in tumor micro environment

We conducted a re-analysis of scRNA-seq data from biopsy samples from 20 LUAD and 22 LUSC patients (GSE148071). Following multiple quality control and filtering steps, a total of 89,887 cells were analyzed for their transcriptomes. Utilizing characteristic canonical cell markers, we identified 13 major cell types, categorized as carcinoma cell types(epithelial cells, ZNF750^low^-TNC ^low^ cancer cells, ZNF750^high^ cancer, TNC^high^ cancer), immune cells (T cells, B lymphocytes, myeloid cells, neutrophils, mast cells, and follicular dendritic cells) and stromal cells(fibroblasts and endothelial cells) (Fig. 4A, Supplemental Figure 6, Supplementary Tables 2 & 3). Notably, in samples from LUSC patients, the proportion of cells exhibiting high ZNF750 expression (15.18%) was significantly higher than that observed in LUAD patients (1.04%), while the proportion of cells with high TNC expression remained relatively stable. This observation suggests that ZNF750 serves as a specific maker for LUSC at the transcriptome level (Fig. 4B-C). Furthermore, consistent with the *in vitro* regulatory ZNF750-TNC axis, a negative correlation between ZNF750 and TNC expression at the single cell transcriptome level was observed (Fig. 4D-E).

**Fig. 6:**
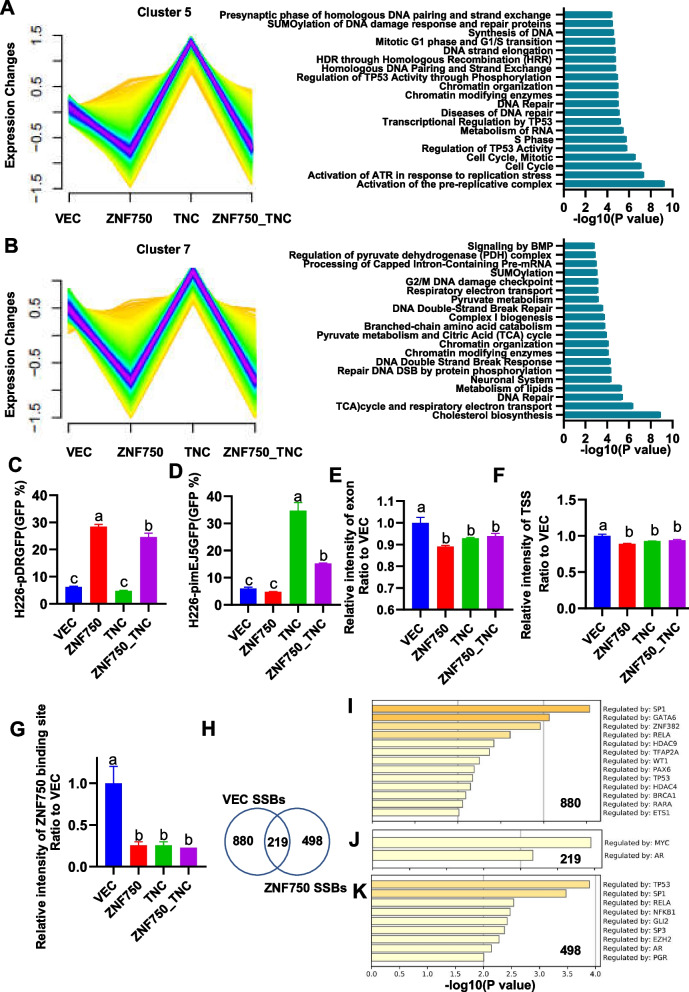
ZNF750-TNC affects DNA damage repair in LUSC cells. **A**-**B** Soft clustering of transcriptome reveals the ZNF750-TNC axis participates the DNA damage repair process analyzed using Mfuzz R package and metascape.org. **C**-**D** DNA damage repair reporter system results. Relative strength of DNA HR repair (**C**) or NHEJ repair (**D**) signal results of three repeated experiments, one-way ANOVA. **E**-**G** DNA SSBs were detected by SSiNGLe method in lung squamous cell carcinoma cells. Ratios of SSBs signal intensities mapped to exons (**E**), transcription start sites (**F**), and ZNF750-binding sites (**G**) in each group versus controls are indicated respectively, one-way ANOVA. **H** Venn diagram of the number of corresponding annotated SSB gene in ZNF750-binding regions; (**I**-**K**) Transcription factor enrichment analysis of venn diagram genes: 880 genes (**I**), 219 genes (**J**), and 498 genes (**K**) respectively, metascape.org. HR: homologous recombination, NHEJ: nonhomologous end joining, TSS: transcription start site, SSBs: single-strand breaks. Significant labels: Different letter means significant difference between groups, same letter means no significant difference between groups; *P* < 0.05 as significant difference

### ZNF750-TNC regulates the immunogenicity of LUSC cells

To explore the dynamics within the tumor microenvironment cell types, we conducted an analysis of cell-cell interactions. Our findings revealed a significant interplay among endothelial cells, fibroblasts, immune cells, and cancer cells (Supplemental Figure 7). Notably, we identified several key ligand-receptor pairs in TNC^high^ cancer cells and immune cells, including NECTIN2-TIGIT, MIF-(CD74+CD44), MDK-NCL, LAMB3-CD44, COL1A2-CD44, COL1A1-CD44, CD99-CD99, APP-CD74, and GZMB-PARD3 were identified in TNC^high^ cancer cells and immune cells (Fig. 5A). In contrast, ZNF750^high^ cancer cells exhibited fewer ligand-receptor interactions. Through TCGA pan-cancer analysis encompassing 33 cancer types, we unveiled significant correlations between immune checkpoint genes, chemokines, chemokine receptors, immune-stimulating and immune-inhibitory genes, and immunity scores with ZNF750 or TNC. Specifically, ZNF750 demonstrated a negative correlation with most immune factors, while TNC showed a positive correlation with the majority of them (Supplemental Figures 8-13). These findings underscore the heightened immunogenicity associated with TNC^high^ cancer cells compared to ZNF750^high^ cancer cells.

**Fig. 7:**
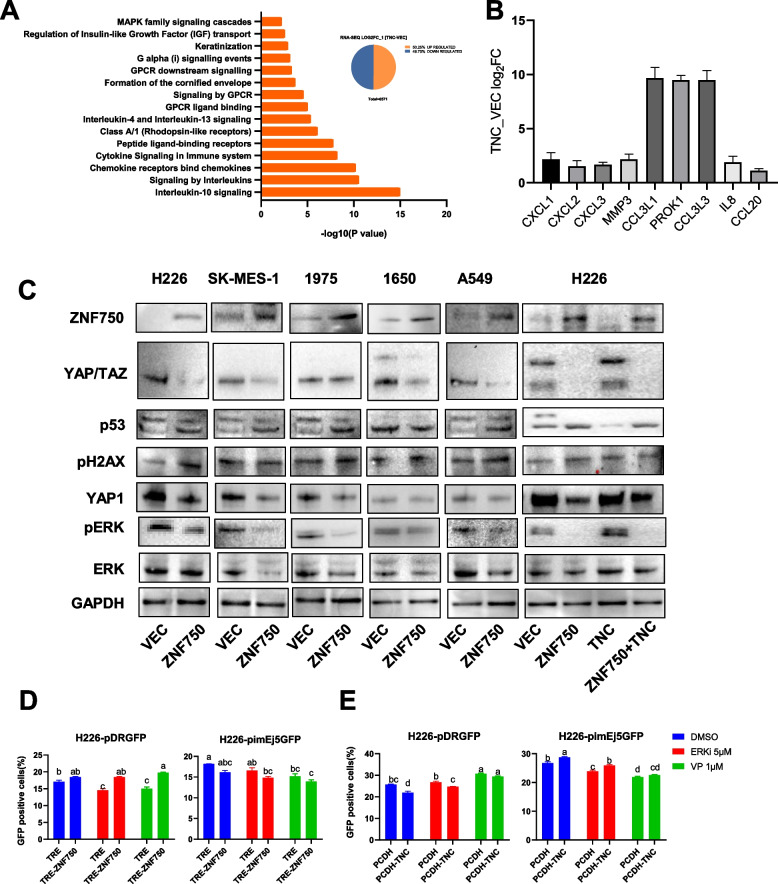
GPCR-YAP/ERK participates the regulation of ZNF750-TNC axis. **A** Top 15 Reactome Gene Sets enrichment analysis results of TNC up-regulated genes in H226 cells. **B** log2FC of genes in cytokine- and chemokine-related GPCR pathway. (**C**)Western blotting of indicated genes in different cell lines. The full-length blots are presented in Supplemental Figures 17-22. **D**-**E** DNA damage repair reporter system results. **D** Relative strength of DNA homologous recombination repair or (**E**) non-homologous end joining signal results, *N* = 3 for each group, twoway ANOVA. VEC: control group transfected with vehicle vector PCDH, ZNF750: ZNF750 overexpression group transfected with PCDH-ZNF750, TNC: TNC overexpression group, ZNF750_TNC: transfected with both PCDH-ZNF750 and PCDH-TNC.TRE: control group transfected with vehicle tet-on vector TRE, TRE-ZNF750: cells transfected with tet-on vector express ZNF750 upon supplied with Doxycycline Hyclate. DMSO: solvents, ERKi: FR180204, VP: Verteporfin. Significant labels: Different letter means significant difference between groups, same letter means no significant difference between groups; *P* < 0.05 as significant difference

Additionally, we established and optimized conditions for stimulating CD8^+^ T cells and NK cells in peripheral blood mononuclear cells (PBMCs) (data not shown). In co-culture experiments involving PBMCs and lung squamous carcinoma cells, immune cell killing assays revealed that ZNF750 overexpression did not significantly enhance the cytotoxicity of PBMCs activated by CD28/CD3/IL-2 against H226 cells at a 5:1 ratio of effector to target (Fig. 5B). Conversely, high expression of TNC substantially increased the sensitivity of H226 cells to activated PBMCs (Fig. 5C).

In summary, ZNF750-mediated suppression of TNC leads to a reduction in the immunogenicity of LUSC cells.

### ZNF750 promotes DNA repair through homologous recombination, whereas TNC promotes non-homologous end joining

We further investigated why the ZNF750-TNC axis affects the malignant phenotypes and immunogenicity of cancer cells. Using temporal-like sequential analysis of transcriptome sequencing results from plasmids control group, ZNF750 overexpression group, TNC overexpression group, and both ZNF750 and TNC overexpression group, we identified clustering of gene expression trends using the Mfuzz R package (Fig. 6A-B). Functional enrichment analysis of genes within cluster5 and cluster7, which exhibited similar trends to *TNC*, revealed the most significant 20 REACTOME terms focusing on DNA damage repair (Fig. 6A-B). Gene Set Enrichment Analysis (GSEA) analysis of ZNF750 or TNC transcriptome sequencing also indicated the involvement of ZNF750-TNC axis in DNA damage repair pathways (Supplementary Table 4). Based on these findings, we hypothesized that the preference between HR and NHEJ may contribute to changes in immunogenicity. Therefore, we evaluated DNA damage response. The results of a HR reporter system (Fig. 6C) and a NHEJ reporter system (Fig. 6D) demonstrated that ZNF750 overexpression up-regulated the HR repair ability of cells while down-regulating NHEJ repair pathway. Additionally, cells overexpressing ZNF750 exhibited increased susceptibility to the apoptosis inducer Staurosporine compared to control cells (Supplemental Figure 14). Conversely, overexpression of TNC down-regulated the repair capacity of cells by HR and up-regulated the NHEJ repair pathway. The observed phenomenon can be explained as follows: Cells tended to prioritize repair via NHEJ strategy, which is characterized by its speed but higher error rate. This preference for NHEJ repair can result in cellular mutations and increased resistance to apoptosis (Supplemental Figure 14). In conclusion, findings from the DNA repair reporter system suggest that ZNF750 facilitates HR repair, while TNC promotes NHEJ repair.

### The single-strand DNA breaks mapped to the ZNF750-binding region in LUSC cells were significantly decreased upon ZNF750 or TNC overexpression

We observed that single-strand DNA "breaktome" (SSBs), reflecting both single-strand breaks and double-strand breaks in DNA, exhibited minimal differences between groups when analyzing SSBs mapped to exon regions or transcription start site (TSS) regions (Fig. 6E-F). However, analysis of SSBs mapped to the ChIP-seq region of ZNF750 , revealed a significant down-regulation in the SSB signal of the ZNF750 overexpression group (25.71%), TNC overexpression group (25.71%), and ZNF750_TNC overexpression group (22.86%) compared to the VEC group (Fig. 6G). This quantitative, single nucleotide resolution evidence indicates that ZNF750-TNC impacts the DNA damage repair process at ZNF750 genome binding sites.

Furthermore, we examined the functions of genes associated with SSB loci in the ZNF750-binding region (Fig.  6H-I).We found that the 880 SSB locus unique to the control group was enriched in transcription factor BRAC1, a key protein involved in HR repair (Fig. 6I) , suggesting compromised HR repair in the control group and accumulation of DNA breaks in the corresponding region. Conversely, the 219 SSB locus appearing in both group was enriched in MYC and AR (Fig. 6J). However, the 498 sites unique to the ZNF750 overexpression group were enriched in EZH2 (Fig. 6K), which is an NHEJ factor known to interfere with HR repair by hindering the formation of RAD51 repair foci [18]. This suggests functional inhibition of the NHEJ pathway in the ZNF750 overexpression group, leading to the accumulation of DNA breaks in the region enriched with NHEJ factors.

Based on these findings, we propose that ZNF750 may play a crucial role in promoting HR repair at DNA damage sites within its genome binding regions.

### The ZNF750-TNC axis significantly affects the expression of key factors in the DNA damage repair related ERK/Hippo signaling pathway

Through the analysis of differentially expressed genes (DEGs) related pathways affected by ZNF750 or TNC overexpression, we observed that both DEGs were enriched in MAPK/ERK signaling pathway (Fig. 2B, Fig. 7A). Specifically, the down-regulated genes at ZNF750 binding sites with ZNF750 expression were enriched in the Hippo signaling pathway (Fig. 2D), while the up-regulated genes overexpressing TNC were enriched in the cytokine- and chemokine-related GPCR pathway (Fig. 7A). Notably, IL-8 (CXCL8), known to induce monocyte recruitment to tumors, was found to be upregulated upon TNC overexpression, along with other pro-proliferative GPCR ligands such as CCL20 [19, 20] (Fig. 7B). This led to an increase in ERK phosphorylation (Fig. 7C), consistent with previous studies linking IL-8-CXCR2-induced phosphorylation of ERK [19, 21].

Furthermore, ZNF750 and TNC influenced the expression of key transcriptional effectors YAP/TAZ in the Hippo signaling pathway. Their influence trended opposite to that of p53 but similar to that of p-ERK (Fig. 7C). Application of YAP or ERK functional inhibitors provided further clarification on the involvement of TNC in regulating DNA damage repair via the Hippo/ERK pathway (Fig. 7D and E). Overall, these findings suggest that the ZNF750-TNC axis significantly influences the Hippo/ERK signaling pathway, thereby playing a role in determining the preference of the DNA damage repair process, whether it involves NHEJ or HR (Fig. 7F).

## Discussion

Numerous insightful studies have been conducted to sensitize immune therapy for non-small cell lung cancer (NSCLC) [22]. In this study, we identified TNC as a downstream regulator of tumor suppressor ZNF750. ZNF750 regulates TNC expression and affects the proliferation, invasion, migration and immunogenicity of cells *in vitro*, as well as tumor growth *in vivo*. Furthermore, our study sheds light on the role of the ZNF750-TNC axis in the regulation of DNA damage repair, which may alter the immunogenicity of cancer cells.

The HR reporter system and NHEJ reporter system demonstrated that ZNF750 overexpression up-regulates the HR repair ability of cells while down-regulating the NHEJ repair pathway. Genome-wide differences in single-strand DNA breaks (SSBs) with ZNF750 or TNC overexpression provide nucleoid resolution maps of DNA damage signals. In genome regions bound by ZNF750, DNA breaks are understood to represent unrepaired areas, potentially due to impaired functionality of transcription factors frequently bound in these regions within the respective experimental groups (see Fig. 6). These observations suggest that the ZNF750-TNC axis influences the distribution of DNA break hotspots and regulates preferences in DNA damage repair between homologous recombination (HR) and non-homologous end joining (NHEJ).

The exploration of pathways influenced by ZNF750-TNC axis suggests its regulatory roles in the Hippo/ERK pathways in LUSC. Hippo/ERK pathways are closely related to the DNA damage repair process [23, 24]. While research on the interaction between ZNF750 and the Hippo pathway mainly focuses on outward migration of epidermal progenitor cells [25, 26], the mechanism study of the regulatory role of ZNF750 in the Hippo pathway in tumor cells is rarely reported. However, recent studies have confirmed that TNC is involved in regulating YAP/TAZ signaling and responses to biomechanical signals [27]. Abnormal expression of TNC can inhibit extracellular matrix adhesion and can activate Hippo signaling [28]. Treatment with YAP/TAZ inhibitor such as Verteporfin [29] can reduce the expression of TNC gene, which is consistent with our research (Fig. 2I and J). Meanwhile, transient overexpression of YAP can upregulate the expression of TNC gene [30]. Moreover, TNC promotes the invasive migration of tumor cells by interacting with integrin α9β1 [30]or α5β1 [31], eliminating actin stress fiber formation, retaining YAP in the cytoplasm and inhibiting the expression of YAP target genes [30]. These studies indicate that TNC may regulate the nuclear localization and transcriptional activity of YAP, forming a feedback regulatory loop of TNC expression.

GPCR regulates Hippo/Erk signaling pathways and can be activated by various cytokines and chemokines to participate in the regulation and response of the tumor immune microenvironment [32, 33]. Overexpression of TNC has been shown to enhance the expression of chemokines such as IL8 (Fig. 7B). There is abundant evidence that IL8-CXCR2 induces phosphorylation of ERK [21], which in turn promotes the expression of YAP/TAZ [34]. Suppression of TNC by ZNF750 and inhibition of ERK or YAP, can increase the HR level and reduce NHEJ in LUSC cells (Figs. 6 and 7). This evidence suggests that the ZNF750-TNC axis plays regulatory roles in the Hippo/ERK signaling pathway and affects DNA damage repair process preference in LUSC.

Importantly, both ZNF750 and TNC are prognostic markers with great potential. The high expression of TNC in clinical samples makes it a suitable target for drug development in LUSC. Moreover, considering that TNC increases immunogenicity of LUSC cells by enhancing NHEJ, it is reasonable to assume that TNC^high^ cancer cells will respond better to immune-related therapies. This hypothesis is supported by the findings in the PBMC killing assay, where the TNC group had the biggest difference at the endpoint of the growth curve (Fig. 5C). These findings suggest that the regulation of LUSC by the ZNF750-TNC axis may be a key factor in its development and can serve as a potential target or biomarker for biotherapeutics, which has a synergistic mechanism of immune cell therapy and is valuable for further *in vivo* research and drug development.

In conclusion, our findings indicate that the regulatory role of the ZNF750-TNC axis plays a crucial role in determining the preference of DNA damage repair through regulation of the Hippo/ERK pathway. This axis determines the choice of cancer cell DNA repair strategy: either the fast but error-prone NHEJ or the slower but more accurate HR. Overall, these findings enhance our understanding of the downstream events with low expression of ZNF750 in LUSC.

### Supplementary Information

**Supplementary Material 1.** 

**Supplementary Material 2.** 

**Supplementary Material 3.** 

**Supplementary Material 4.** 

**Supplementary Material 5.** 

**Supplementary Material 6.** 

## Data Availability

The data that support the findings of this study are available from the corresponding author upon reasonable request. Next generation sequencing data have been deposited into CNGB Sequence Archive (CNSA) of China National GeneBank DataBase (CNGBdb) with accession number CNP [CNP0001514].
