# Supplementary material for: Tenascin C as a novel zinc finger protein 750 target regulating the immunogenicity via DNA damage in lung squamous cell carcinoma
Source: BMC Cancer. 2024 May 6;24:561. doi: 10.1186/s12885-024-12285-8 (PMC11071264; doi:10.1186/s12885-024-12285-8)
Supplement: Supplementary file 1 — Supplementary Material 1. [file 12885_2024_12285_MOESM1_ESM.pdf]

Tenascin C as a Novel Zinc Finger Protein 750 Target Regulating the Immunogenicity via DNA Damage in Lung Squamous Cell Carcinoma

Supplementary Figures & Figure Legends:

A

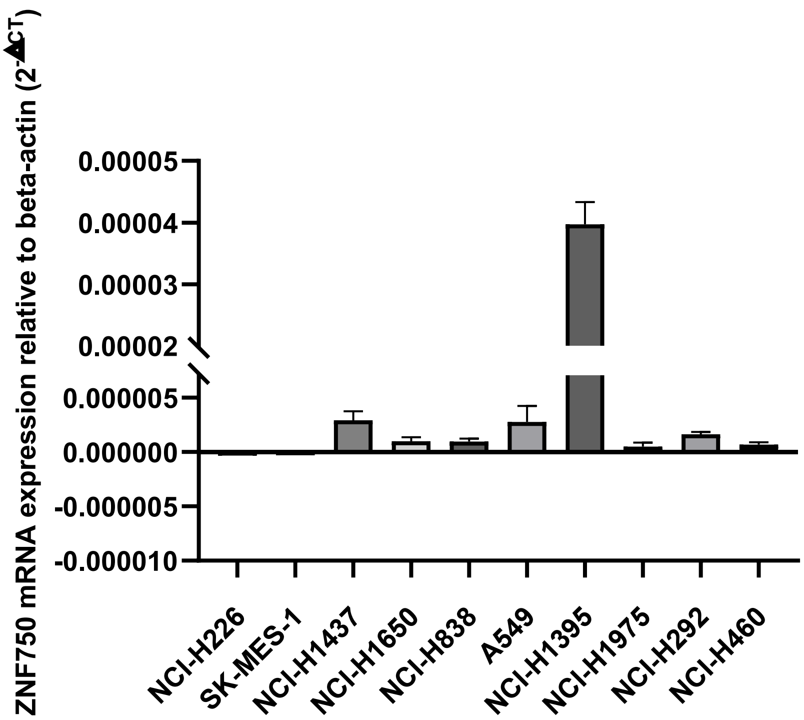

ZNF750 mRNA expression relative to beta-actin (2-ΔCT)
0.00005
0.00004
0.00003
0.00002
0.000005
0.000000
-0.000005
-0.000010
NCI-H226
SK-MES-1
NCI-H1437
NCI-H1650
NCI-H838
A549
NCI-H1395
NCI-H1975
NCI-H292
NCI-H460

B

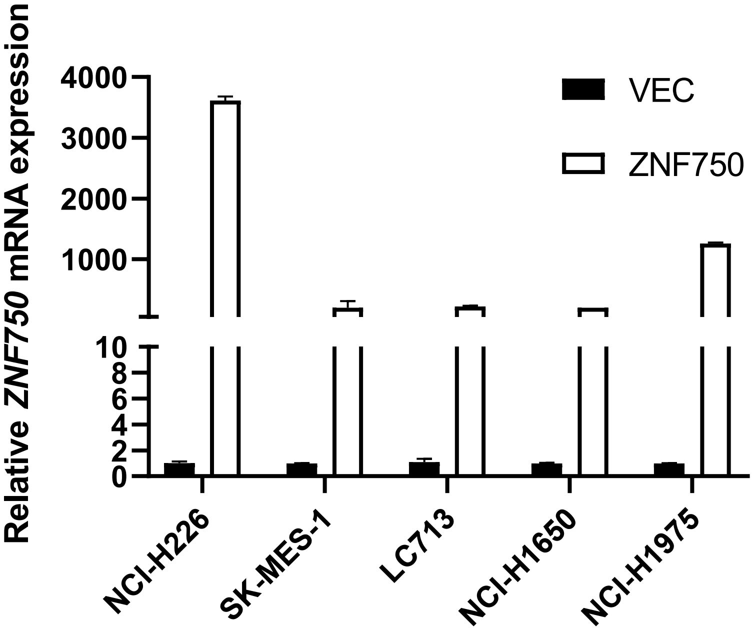

Relative ZNF750 mRNA expression
4000
3000
2000
1000
10
8
6
4
2
0
VEC
ZNF750
NCI-H226
SK-MES-1
LC713
NCI-H1650
NCI-H1975

Supplemental Figure 1

ZNF750 RNA expression in cells. (A) Relative ZNF750 mRNA expression in different lung cancer cell lines. (B) Validation of ZNF750 expression in five indicated cell lines transfected with ZNF750 overexpression plasmids or vehicle plasmid, *N* = 3 for each group.

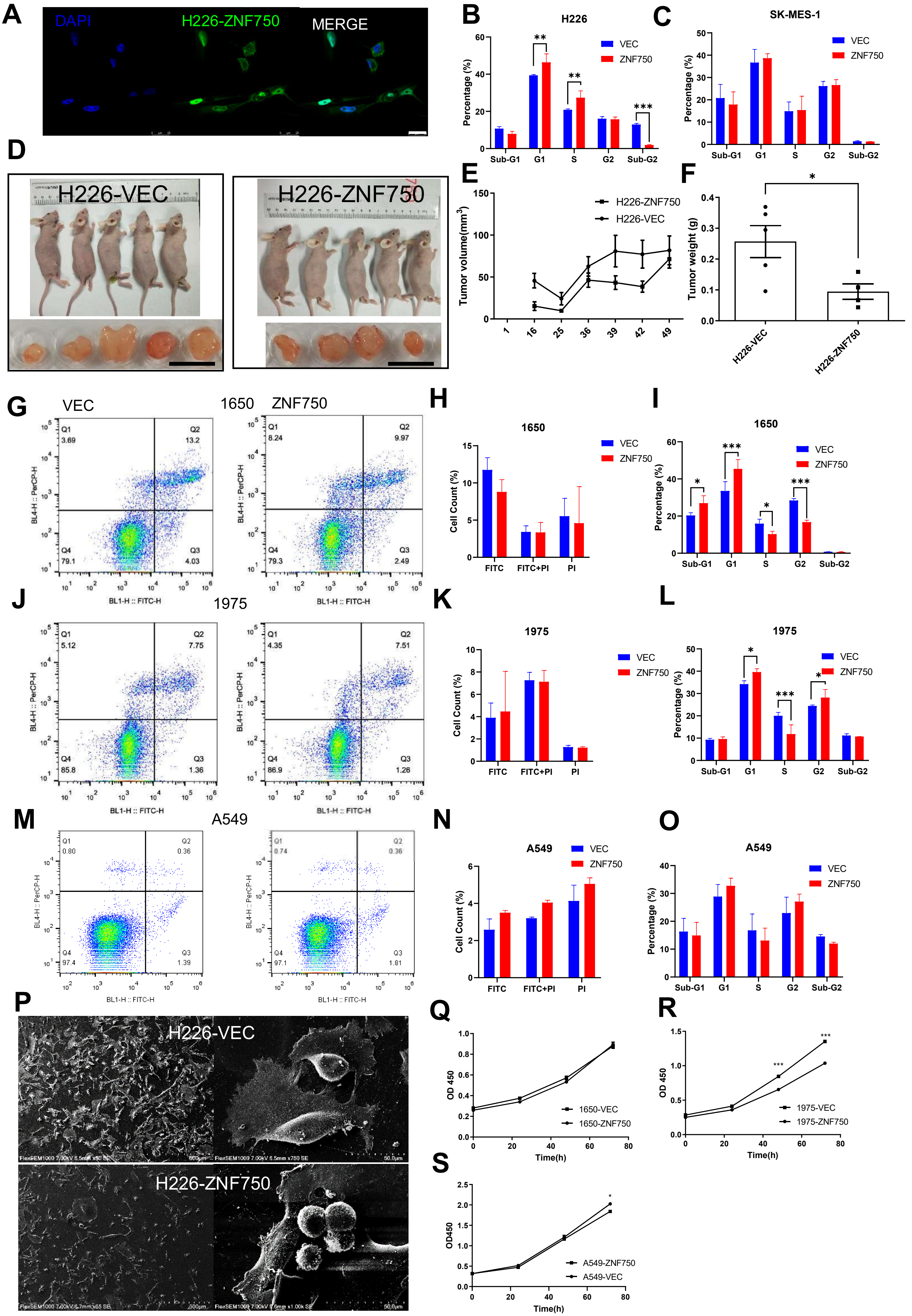
A
DAPI
H226-ZNF750
MERGE
B
H226
VEC
ZNF750
Percentage (%)
Sub-G1
G1
S
G2
Sub-G2
C
SK-MES-1
D
H226-VEC
H226-ZNF750
E
Tumor volume(mm3)
H226-ZNF750
H226-VEC
F
Tumor weight (g)
G
VEC
1650
ZNF750
BL4-H :: PerCP-H
BL1-H :: FITC-H
H
1650
Cell Count (%)
FITC
FITC+PI
PI
I
1650
J
1975
K
1975
L
1975
M
A549
N
A549
O
A549
P
H226-VEC
H226-ZNF750
Q
OD 450
Time(h)
1650-VEC
1650-ZNF750
R
1975-VEC
1975-ZNF750
S
OD450
A549-ZNF750
A549-VEC

Supplemental Figure 2

Phenotypic changes in different lung cancer cell lines with ZNF750 overexpression. (A) ZNF750 was overexpressed in H226 cells, Immunefluorescence shows the nuclear localization of ZNF750, scale bar = 25 μm. (B-C) Cell cycle analysis in LUSC cells, multiple *t* test. (D-F) overexpression of ZNF750 in lung squamous cell carcinoma cell lines significantly slowed tumor growth in nude mice (*N* = 5 for each group): (D) scale bar = 1 cm in, (E) tumor growth curve, two-way ANOVA. (F)Tumor weight at day 49, two sample *t* test. (G-O) Apoptosis analysis and cell cycle analysis in different lung cancer cell lines: (G-I)1650, (J-L)1975, (M-O) A549, multiple *t* test. (P) Scanning Electron Microscopy result of H226 cells, scale bar = 500 μm(left panel) or 50 μm(right panel). (Q-S) Cell proliferation analysis in different lung cancer cell lines: (Q)1650, (R)1975, (S)A549. *: $P < 0.05$, **: $P < 0.01$, ***: $P < 0.001$.

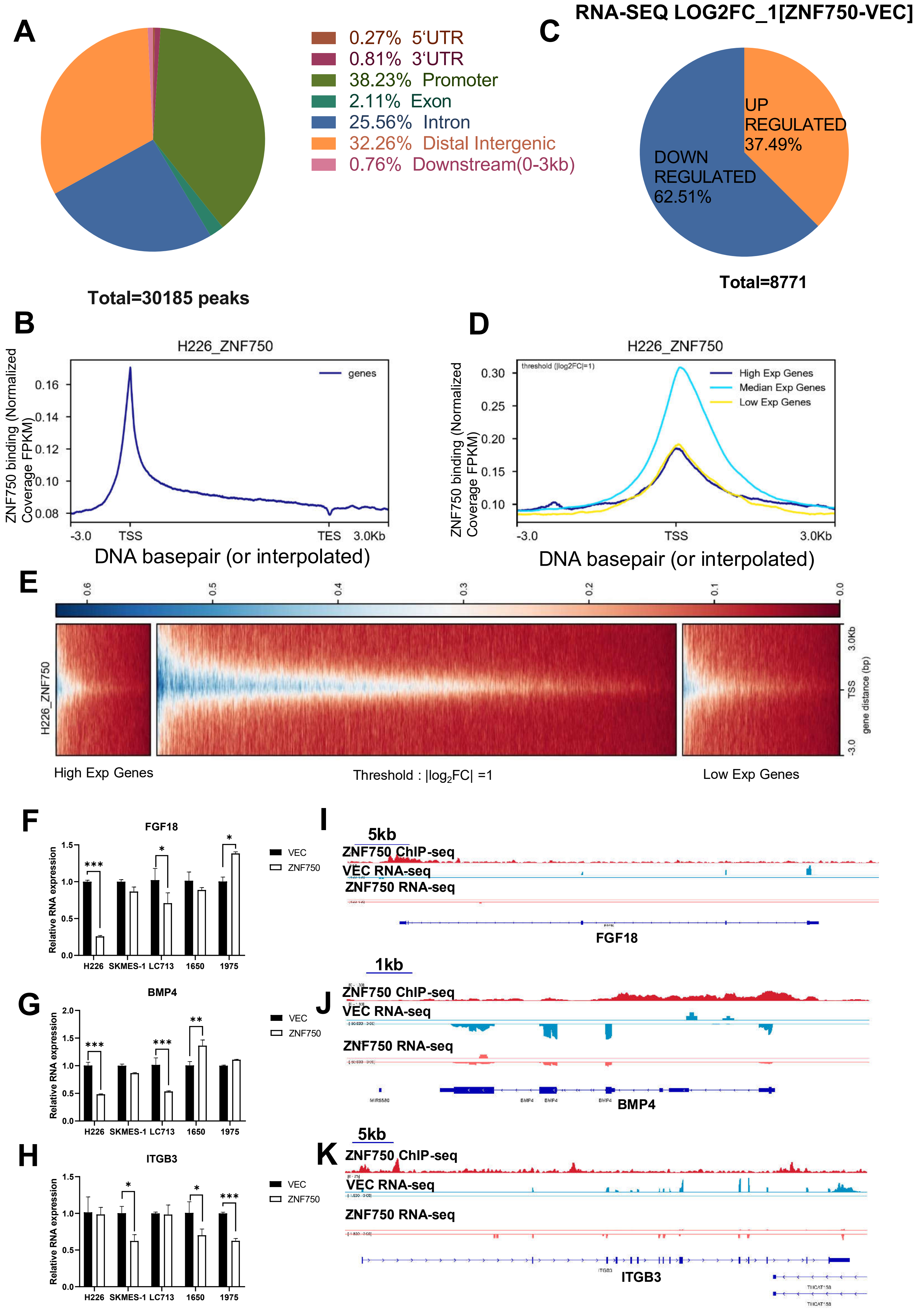

A
0.27% 5‘UTR
0.81% 3‘UTR
38.23% Promoter
2.11% Exon
25.56% Intron
32.26% Distal Intergenic
0.76% Downstream(0-3kb)
Total=30185 peaks
C
RNA-SEQ LOG2FC_1[ZNF750-VEC]
UP REGULATED 37.49%
DOWN REGULATED 62.51%
Total=8771
B
H226_ZNF750
genes
ZNF750 binding (Normalized Coverage FPKM)
-3.0 TSS TES 3.0Kb
DNA basepair (or interpolated)
D
H226_ZNF750
threshold (|log2FC|=1)
High Exp Genes
Median Exp Genes
Low Exp Genes
ZNF750 binding (Normalized Coverage FPKM)
-3.0 TSS 3.0Kb
DNA basepair (or interpolated)
E
H226_ZNF750
3.0Kb TSS -3.0 gene distance (bp)
High Exp Genes
Threshold : |log2FC| =1
Low Exp Genes
F
FGF18
G
BMP4
H
ITGB3
Relative RNA expression
VEC
ZNF750
H226 SKMES-1 LC713 1650 1975
I
5kb
ZNF750 ChIP-seq
VEC RNA-seq
ZNF750 RNA-seq
FGF18
J
1kb
BMP4
K
5kb
ITGB3

Supplemental Figure 3

ZNF750 binding sites in H226 cells. (A) Pie plot of ZNF750 binding regions distribution.（B）ZNF750 binding in H226 across gene regions genome-wide as visualized by deeptools (https://deeptools.readthedocs.io/en/develop/). Binding intensity at upstream promoters, TSSs, gene bodies, and transcription end sites (TESs) are shown in Normalized Coverage FPKM. (C) Pie plot of RNA-seq differential expressed genes with ZNF750 overexpression. (D)Correlation of ZNF750 binding by ChIP-seq (displayed by deeptools) with gene expression levels by RNA-seq shows that, with ZNF750 expression, genes expressed at median levels display dramatically higher enrichment of ZNF750. (E) Peak heatmap of ZNF750 grouped by gene expression levels. The threshold of RNA-seq is $|log_2FC|=1$. (F-K) IGV views and RT-qPCR results of ZNF750 binding candidate genes. RT-qPCR results of (F)FGF18, (G)BMP4, and (H)ITGB3, and IGV views of indicated ChIP-seq and RNA-seq results in (I)FGF18, (J)BMP4, and (K)ITGB3. Related to Figure 2.

A

H&E

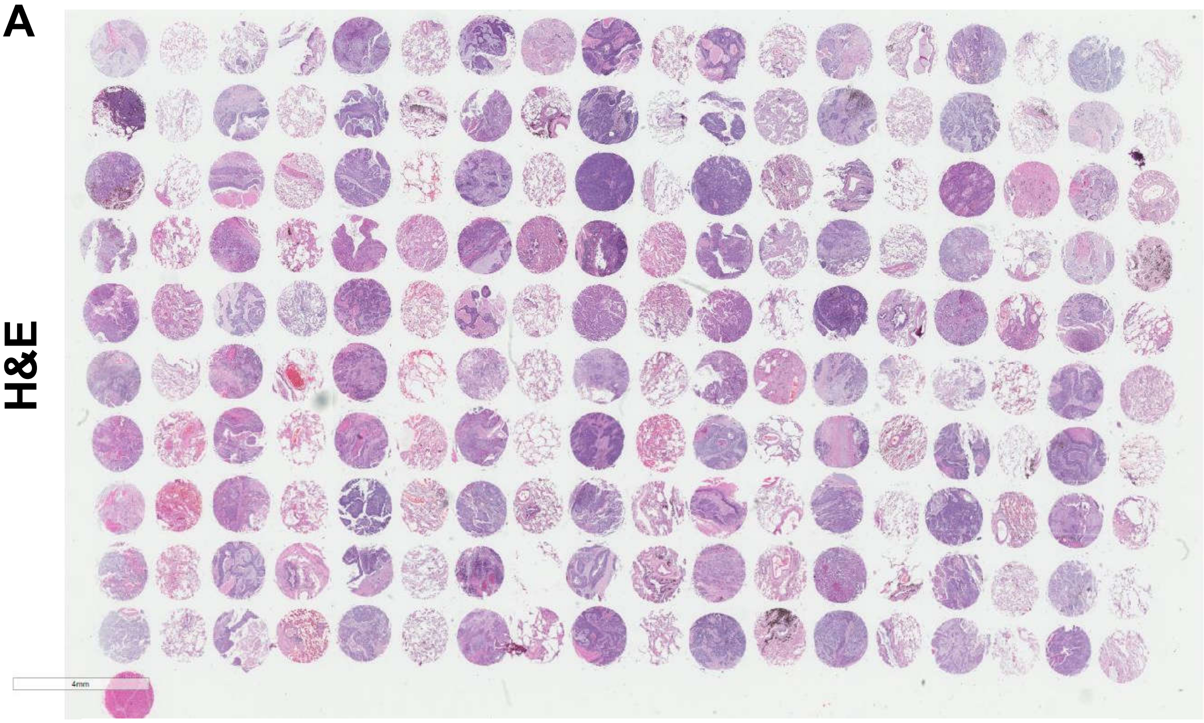

B

ZNF750 IHC

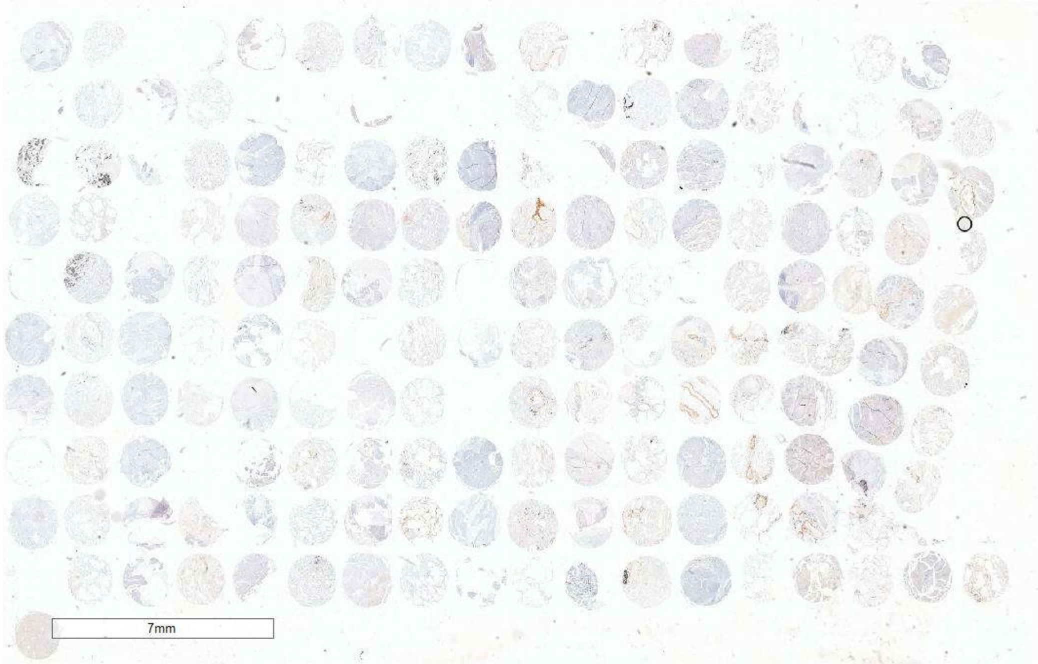

C

TNC IHC

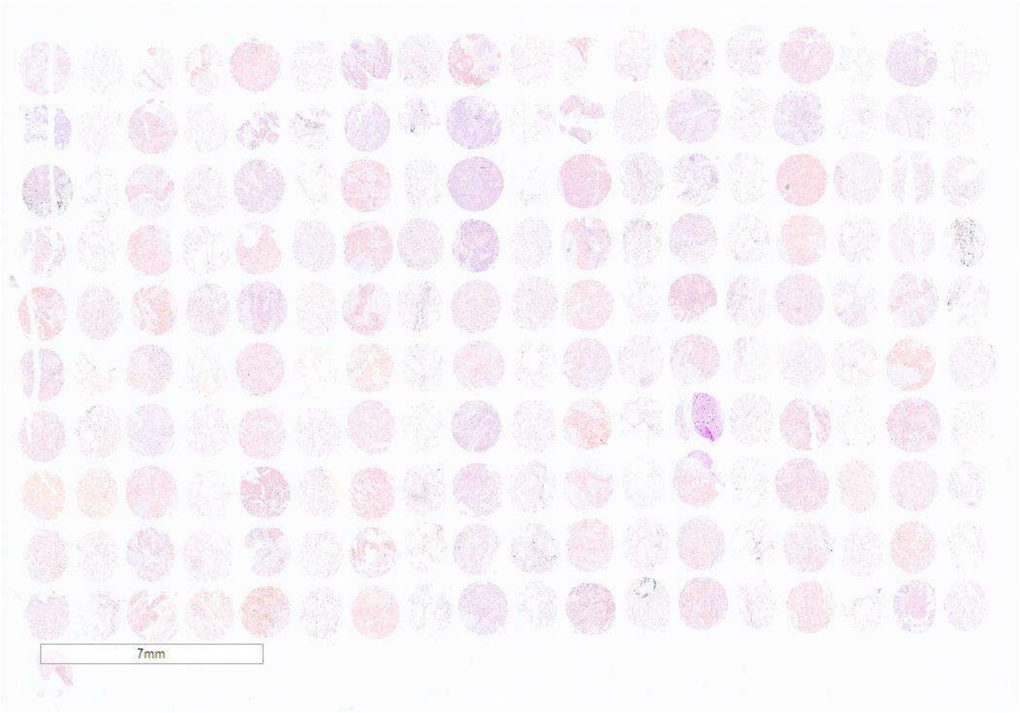

Supplemental Figure 4

An overview of LUSC patient tissue arrays. (A)Hematoxylin and eosin (HE) staining, scale bar = 4 mm (B-C) Immunohistochemistry of ZNF750(B) and TNC(C), scale bar = 7 mm.

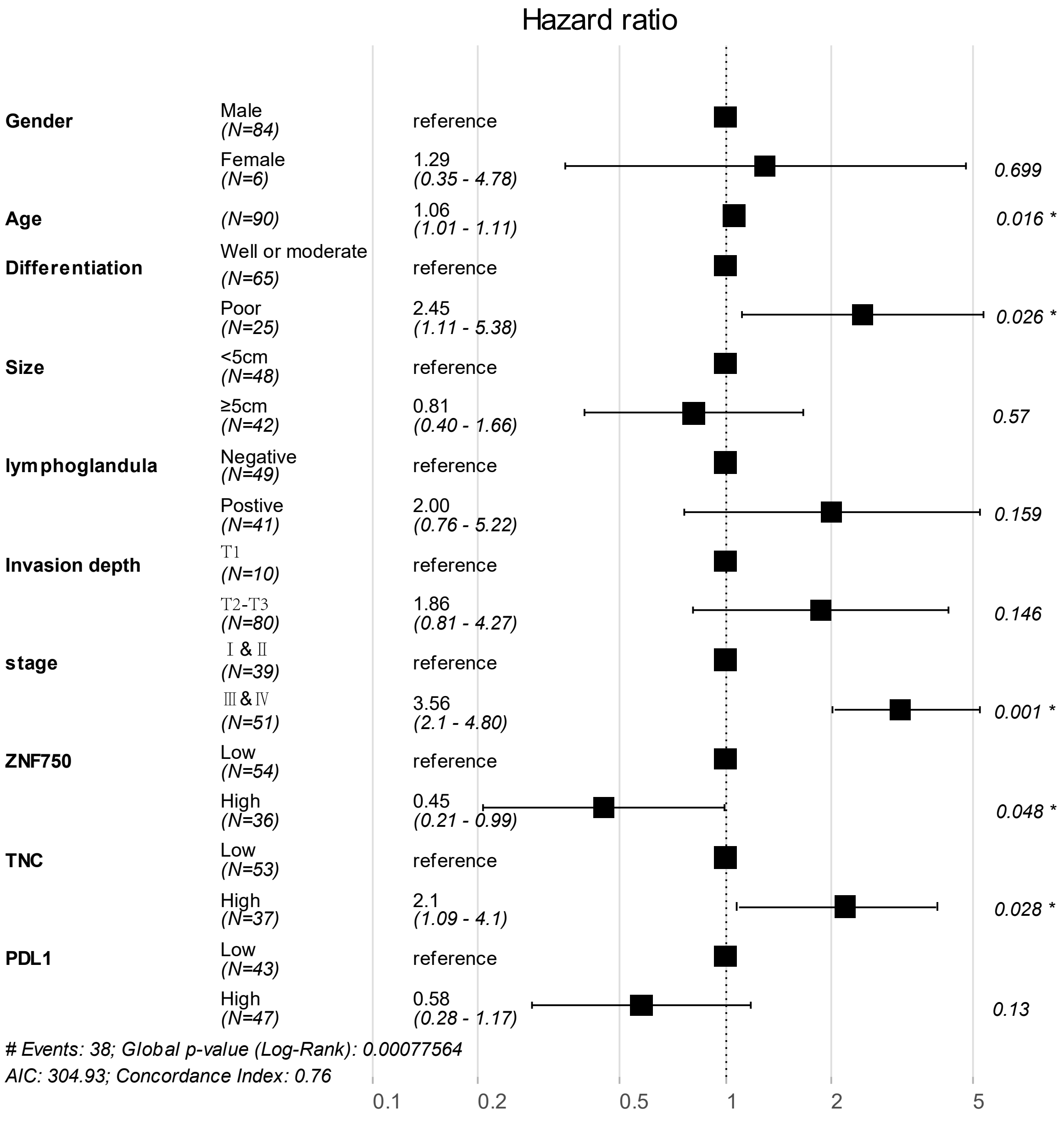

Hazard ratio
Gender
Male (N=84)
reference
Female (N=6)
1.29 (0.35 - 4.78)
0.699
Age
(N=90)
1.06 (1.01 - 1.11)
0.016 *
Differentiation
Well or moderate (N=65)
reference
Poor (N=25)
2.45 (1.11 - 5.38)
0.026 *
Size
<5cm (N=48)
reference
≥5cm (N=42)
0.81 (0.40 - 1.66)
0.57
lymphoglandula
Negative (N=49)
reference
Postive (N=41)
2.00 (0.76 - 5.22)
0.159
Invasion depth
T1 (N=10)
reference
T2-T3 (N=80)
1.86 (0.81 - 4.27)
0.146
stage
Ⅰ&Ⅱ (N=39)
reference
Ⅲ&Ⅳ (N=51)
3.56 (2.1 - 4.80)
0.001 *
ZNF750
Low (N=54)
reference
High (N=36)
0.45 (0.21 - 0.99)
0.048 *
TNC
Low (N=53)
reference
High (N=37)
2.1 (1.09 - 4.1)
0.028 *
PDL1
Low (N=43)
reference
High (N=47)
0.58 (0.28 - 1.17)
0.13
Events: 38; Global p-value (Log-Rank): 0.00077564
AIC: 304.93; Concordance Index: 0.76
0.1
0.2
0.5
1
2
5

Supplemental Figure 5

Forest plot portraying the hazard ratio (HR) and 95% CI of overall survival in IHC cohort. Strata were gender (female or male), age, differentiation (well or moderate or poor), tumor size (5cm as boundary), lymphoglandula metastasis (negative or positive), invasion depth (no or yes), stage (I&II or III&IV), ZNF750/TNC/PD-L1(low expression or high expression). using ggforestplot R package(Version 1.0). *: $P < 0.05$.

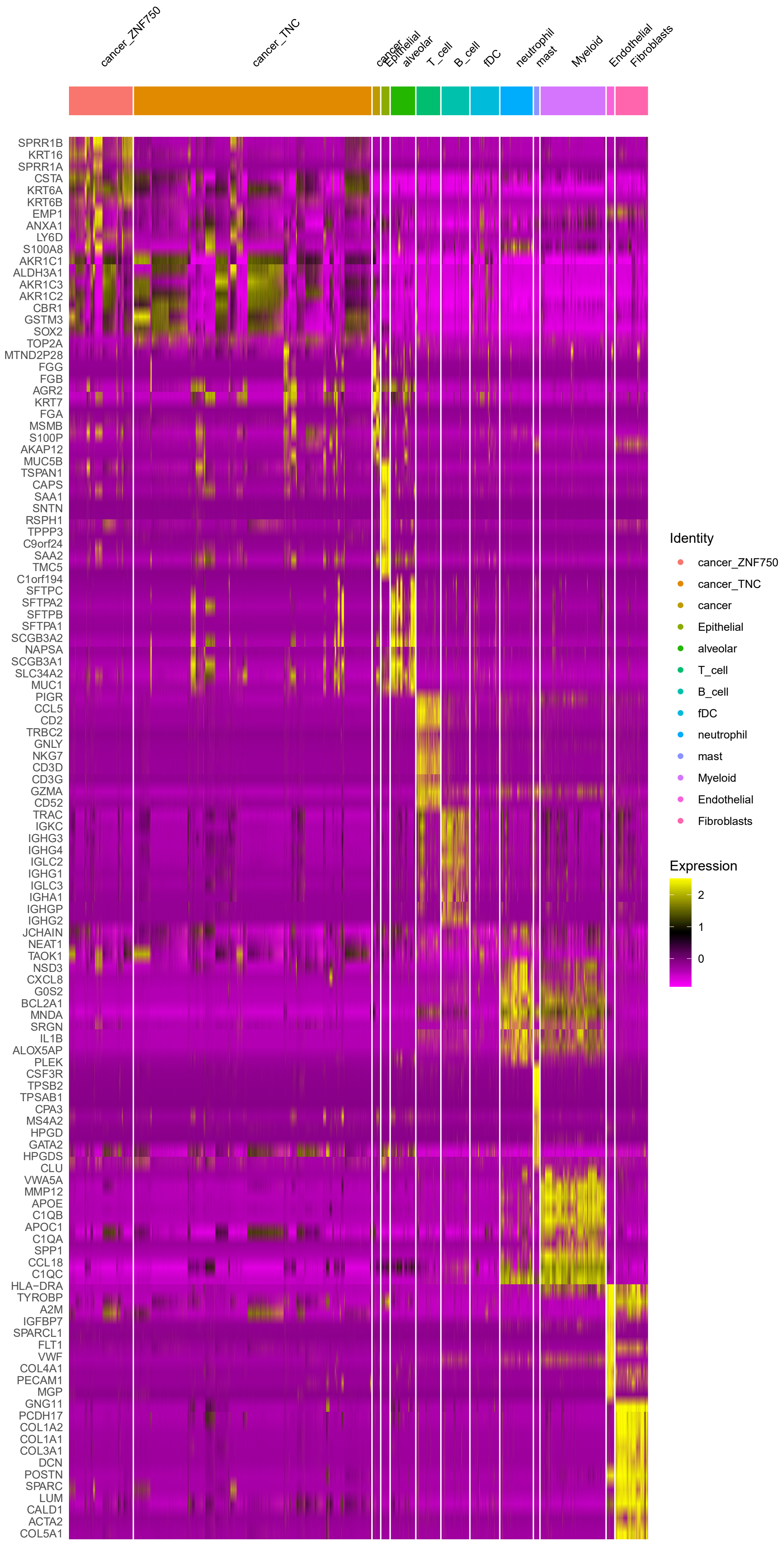
cancer_ZNF750
cancer_TNC
cancer
Epithelial
alveolar
T_cell
B_cell
fDC
neutrophil
mast
Myeloid
Endothelial
Fibroblasts
SPRR1B
KRT16
SPRR1A
CSTA
KRT6A
KRT6B
EMP1
ANXA1
LY6D
S100A8
AKR1C1
ALDH3A1
AKR1C3
AKR1C2
CBR1
GSTM3
SOX2
TOP2A
MTND2P28
FGG
FGB
AGR2
KRT7
FGA
MSMB
S100P
AKAP12
MUC5B
TSPAN1
CAPS
SAA1
SNTN
RSPH1
TPPP3
C9orf24
SAA2
TMC5
C1orf194
SFTPC
SFTPA2
SFTPB
SFTPA1
SCGB3A2
NAPSA
SCGB3A1
SLC34A2
MUC1
PIGR
CCL5
CD2
TRBC2
GNLY
NKG7
CD3D
CD3G
GZMA
CD52
TRAC
IGKC
IGHG3
IGHG4
IGLC2
IGHG1
IGLC3
IGHA1
IGHGP
IGHG2
JCHAIN
NEAT1
TAOK1
NSD3
CXCL8
G0S2
BCL2A1
MNDA
SRGN
IL1B
ALOX5AP
PLEK
CSF3R
TPSB2
TPSAB1
CPA3
MS4A2
HPGD
GATA2
HPGDS
CLU
VWA5A
MMP12
APOE
C1QB
APOC1
C1QA
SPP1
CCL18
C1QC
HLA-DRA
TYROBP
A2M
IGFBP7
SPARCL1
FLT1
VWF
COL4A1
PECAM1
MGP
GNG11
PCDH17
COL1A2
COL1A1
COL3A1
DCN
POSTN
SPARC
LUM
CALD1
ACTA2
COL5A1
Identity
cancer_ZNF750
cancer_TNC
cancer
Epithelial
alveolar
T_cell
B_cell
fDC
neutrophil
mast
Myeloid
Endothelial
Fibroblasts
Expression
2
1
0

Supplemental Figure 6

Heatmap of canonical cell-type markers of 13 major cell types.

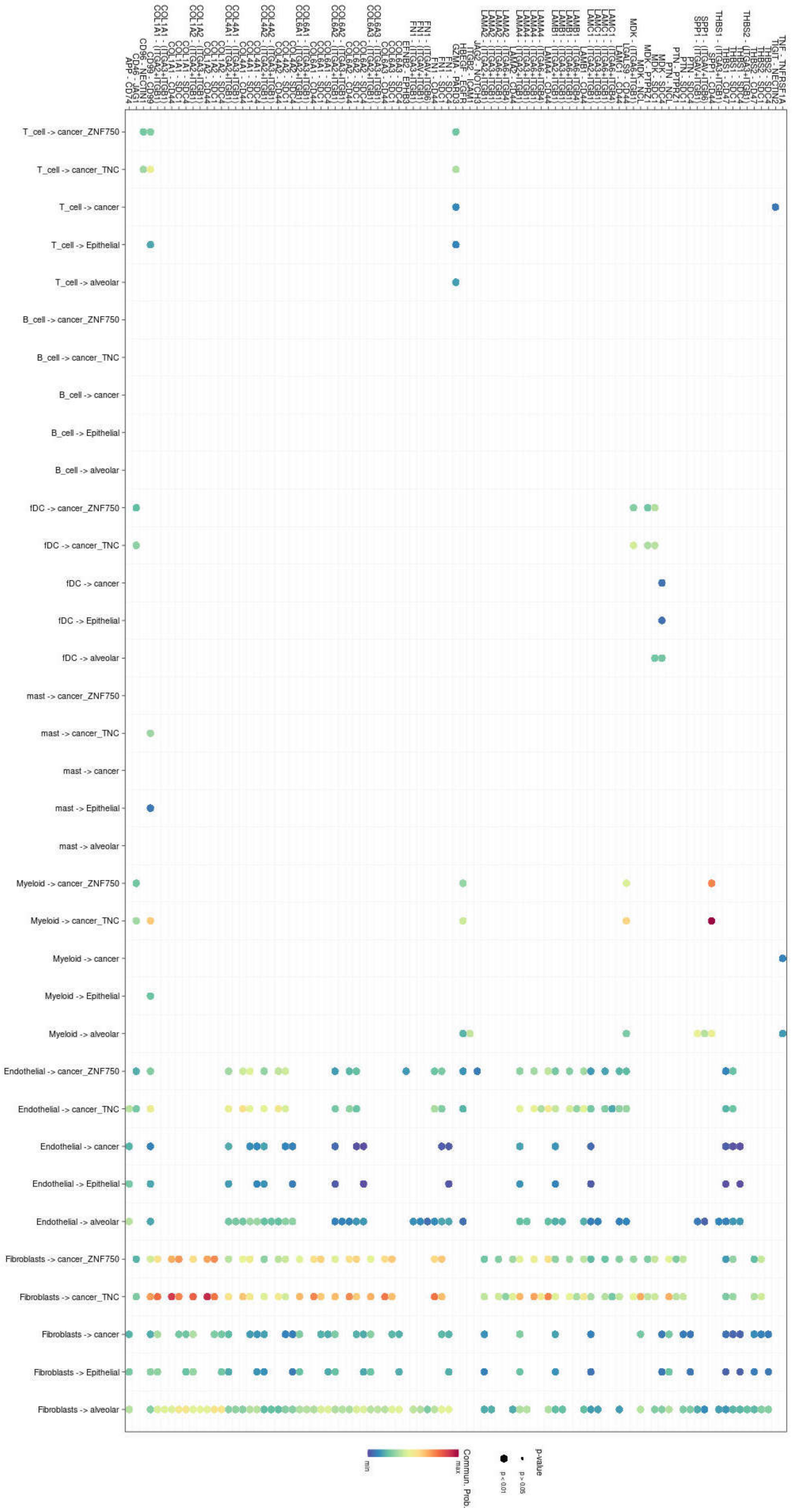

T_cell -> cancer_ZNF750
T_cell -> cancer_TNC
T_cell -> cancer
T_cell -> Epithelial
T_cell -> alveolar
B_cell -> cancer_ZNF750
B_cell -> cancer_TNC
B_cell -> cancer
B_cell -> Epithelial
B_cell -> alveolar
fDC -> cancer_ZNF750
fDC -> cancer_TNC
fDC -> cancer
fDC -> Epithelial
fDC -> alveolar
mast -> cancer_ZNF750
mast -> cancer_TNC
mast -> cancer
mast -> Epithelial
mast -> alveolar
Myeloid -> cancer_ZNF750
Myeloid -> cancer_TNC
Myeloid -> cancer
Myeloid -> Epithelial
Myeloid -> alveolar
Endothelial -> cancer_ZNF750
Endothelial -> cancer_TNC
Endothelial -> cancer
Endothelial -> Epithelial
Endothelial -> alveolar
Fibroblasts -> cancer_ZNF750
Fibroblasts -> cancer_TNC
Fibroblasts -> cancer
Fibroblasts -> Epithelial
Fibroblasts -> alveolar
Commun. Prob.
min
max
p-value
p > 0.05
p < 0.01

Supplemental Figure 7

Growth factors between cancer cells and endothelial cells, fibroblasts, immune cells.

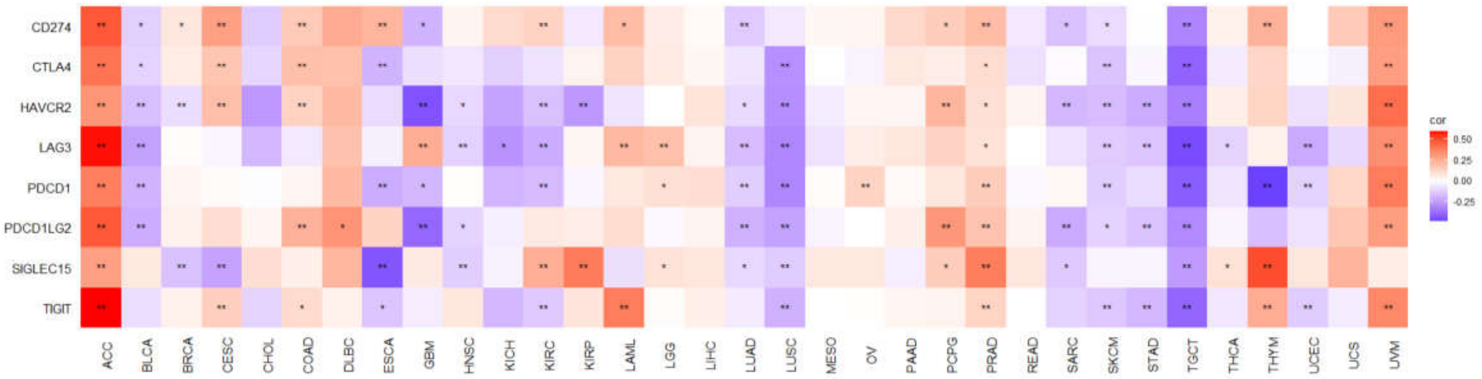
CD274
CTLA4
HAVCR2
LAG3
PDCD1
PDCD1LG2
SIGLEC15
TIGIT
ACC BLCA BRCA CESC CHOL COAD DLBC ESCA GBM HNSC KICH KIRC KIRP LAML LGG LIHC LUAD LUSC MESO OV PAAD PCPG PRAD READ SARC SKCM STAD TGCT THCA THYM UCEC UCS UVM
cor
0.50
0.25
0.00
-0.25

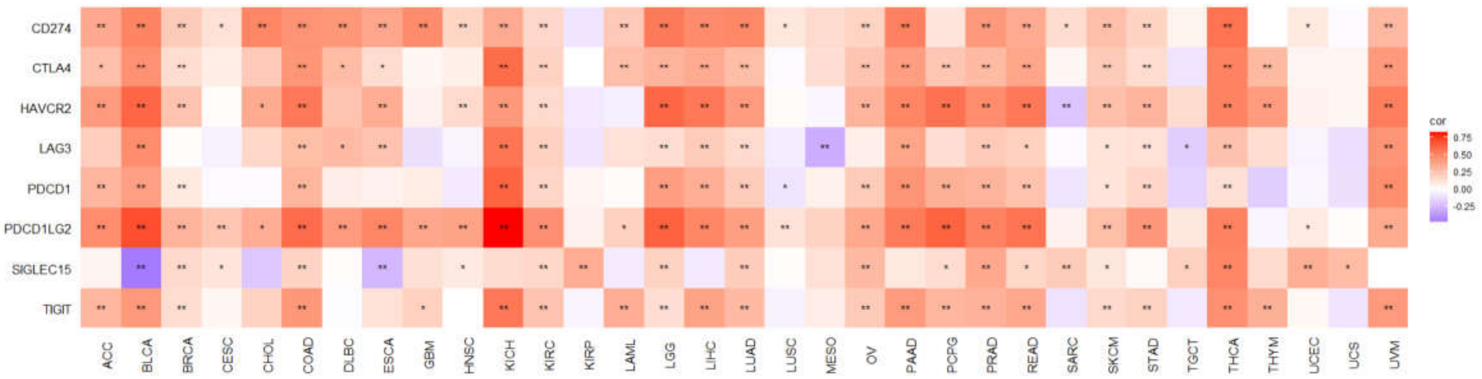
CD274
CTLA4
HAVCR2
LAG3
PDCD1
PDCD1LG2
SIGLEC15
TIGIT
ACC BLCA BRCA CESC CHOL COAD DLBC ESCA GBM HNSC KICH KIRC KIRP LAML LGG LIHC LUAD LUSC MESO OV PAAD PCPG PRAD READ SARC SKCM STAD TGCT THCA THYM UCEC UCS UVM
cor
0.75
0.50
0.25
0.00
-0.25

Supplemental Figure 8

The pan-cancer ICGs correlation analysis demonstrates the expression level correlation between immune checkpoint genes and either ZNF750 (upper panel) or TNC (lower panel) in 33 types of cancer. This analysis was conducted using the TCGA-plot R package.

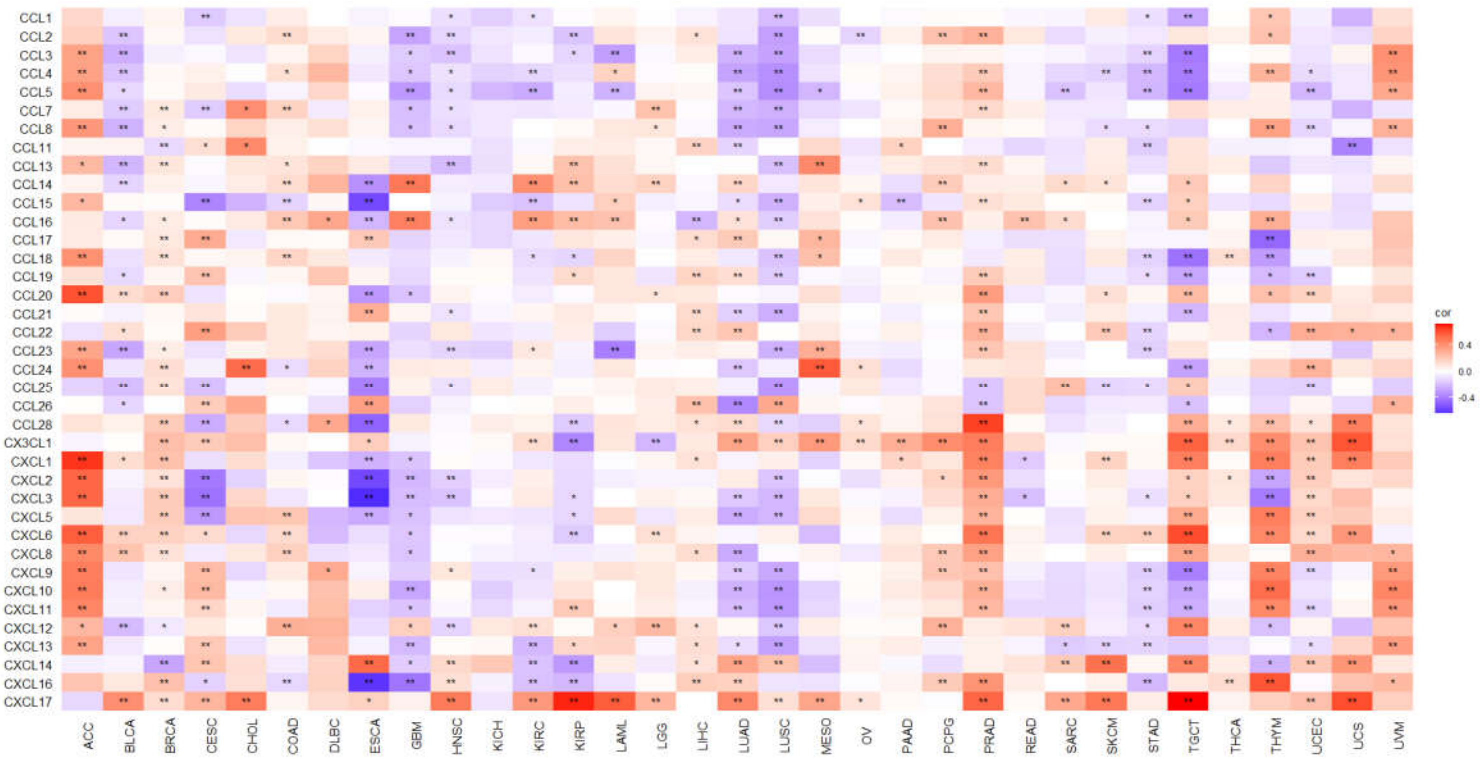
CCL1
CCL2
CCL3
CCL4
CCL5
CCL7
CCL8
CCL11
CCL13
CCL14
CCL15
CCL16
CCL17
CCL18
CCL19
CCL20
CCL21
CCL22
CCL23
CCL24
CCL25
CCL26
CCL28
CX3CL1
CXCL1
CXCL2
CXCL3
CXCL5
CXCL6
CXCL8
CXCL9
CXCL10
CXCL11
CXCL12
CXCL13
CXCL14
CXCL16
CXCL17
ACC
BLCA
BRCA
CESC
CHOL
COAD
DLBC
ESCA
GBM
HNSC
KICH
KIRC
KIRP
LAML
LGG
LIHC
LUAD
LUSC
MESO
OV
PAAD
PCPG
PRAD
READ
SARC
SKCM
STAD
TGCT
THCA
THYM
UCEC
UCS
UVM
cor
0.4
0.0
-0.4

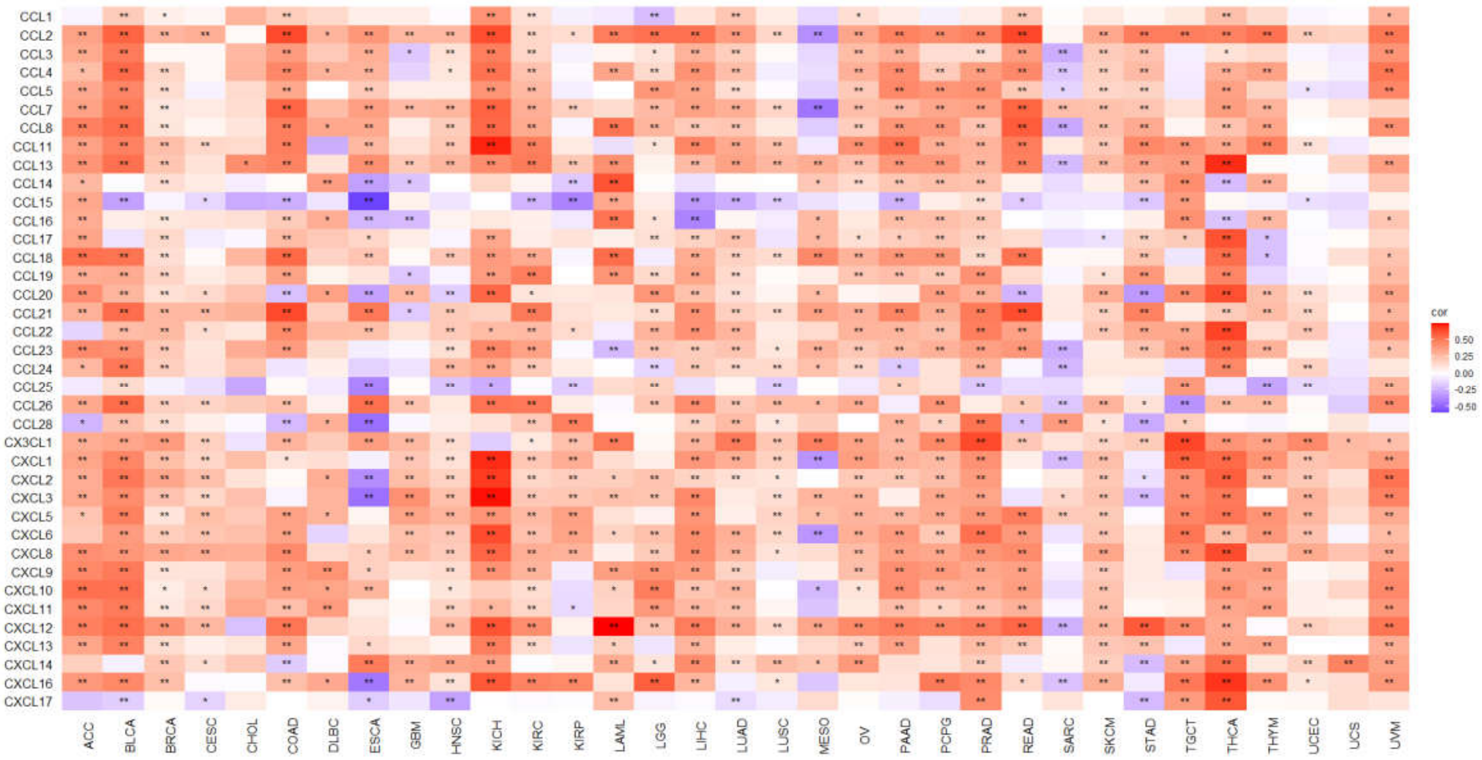
CCL1
CCL2
CCL3
CCL4
CCL5
CCL7
CCL8
CCL11
CCL13
CCL14
CCL15
CCL16
CCL17
CCL18
CCL19
CCL20
CCL21
CCL22
CCL23
CCL24
CCL25
CCL26
CCL28
CX3CL1
CXCL1
CXCL2
CXCL3
CXCL5
CXCL6
CXCL8
CXCL9
CXCL10
CXCL11
CXCL12
CXCL13
CXCL14
CXCL16
CXCL17
ACC
BLCA
BRCA
CESC
CHOL
COAD
DLBC
ESCA
GBM
HNSC
KICH
KIRC
KIRP
LAML
LGG
LIHC
LUAD
LUSC
MESO
OV
PAAD
PCPG
PRAD
READ
SARC
SKCM
STAD
TGCT
THCA
THYM
UCEC
UCS
UVM
cor
0.50
0.25
0.00
-0.25
-0.50

Supplemental Figure 9

The pan-cancer ICGs correlation analysis demonstrates the expression level correlation between chemokine genes and either ZNF750 (upper panel) or TNC (lower panel) in 33 types of cancer. This analysis was conducted using the TCGA-plot R package.

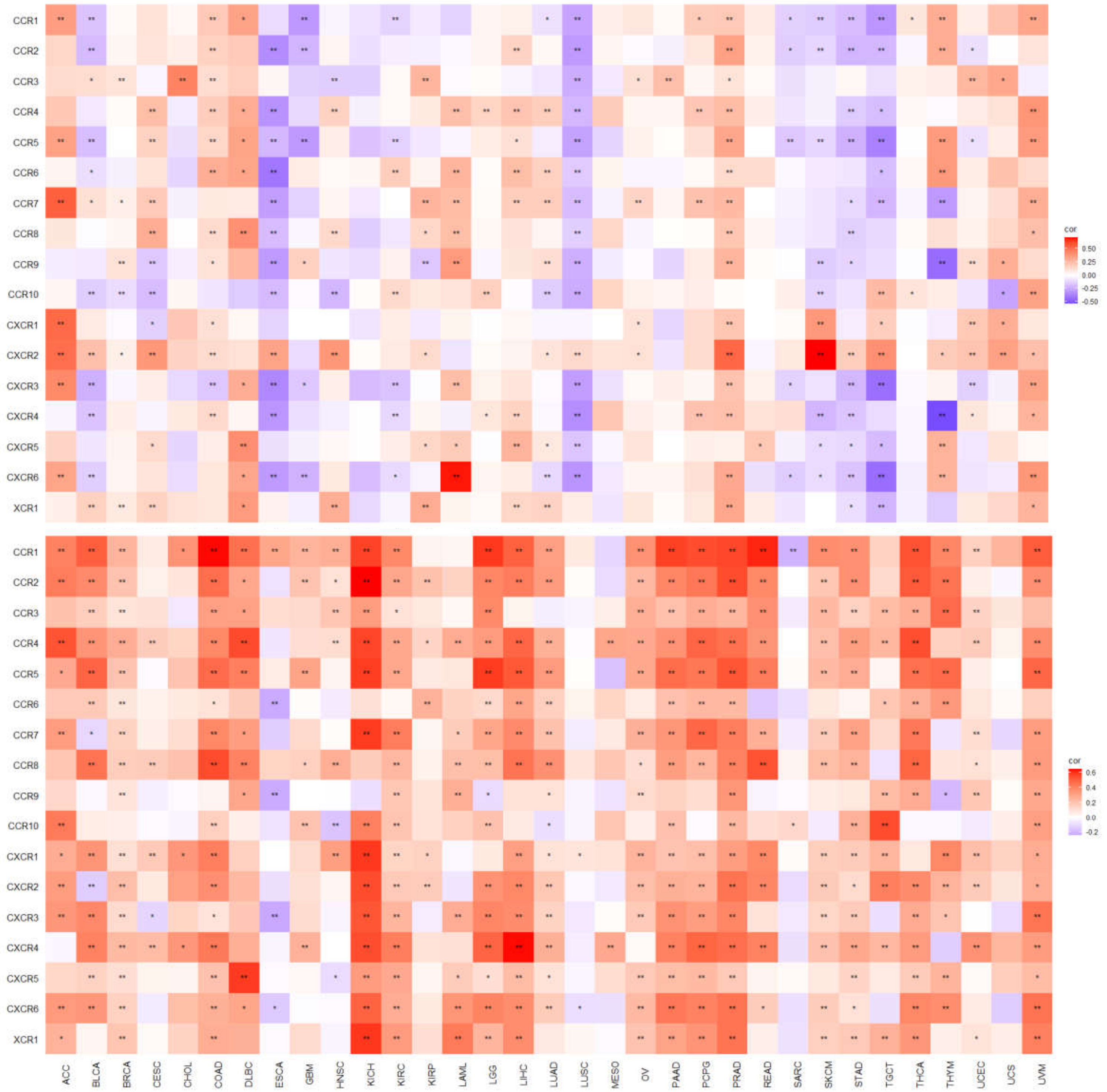

CCR1
CCR2
CCR3
CCR4
CCR5
CCR6
CCR7
CCR8
CCR9
CCR10
CXCR1
CXCR2
CXCR3
CXCR4
CXCR5
CXCR6
XCR1
cor
0.50
0.25
0.00
-0.25
-0.50
cor
0.6
0.4
0.2
0.0
-0.2
ACC
BLCA
BRCA
CESC
CHOL
COAD
DLBC
ESCA
GBM
HNSC
KICH
KIRC
KIRP
LAML
LGG
LIHC
LUAD
LUSC
MESO
OV
PAAD
PCPG
PRAD
READ
SARC
SKCM
STAD
TGCT
THCA
THYM
UCEC
UCS
UVM

Supplemental Figure 10

The pan-cancer ICGs correlation analysis demonstrates the expression level correlation between chemokine receptors genes and either ZNF750 (upper panel) or TNC (lower panel) in 33 types of cancer. This analysis was conducted using the TCGA-plot R package.

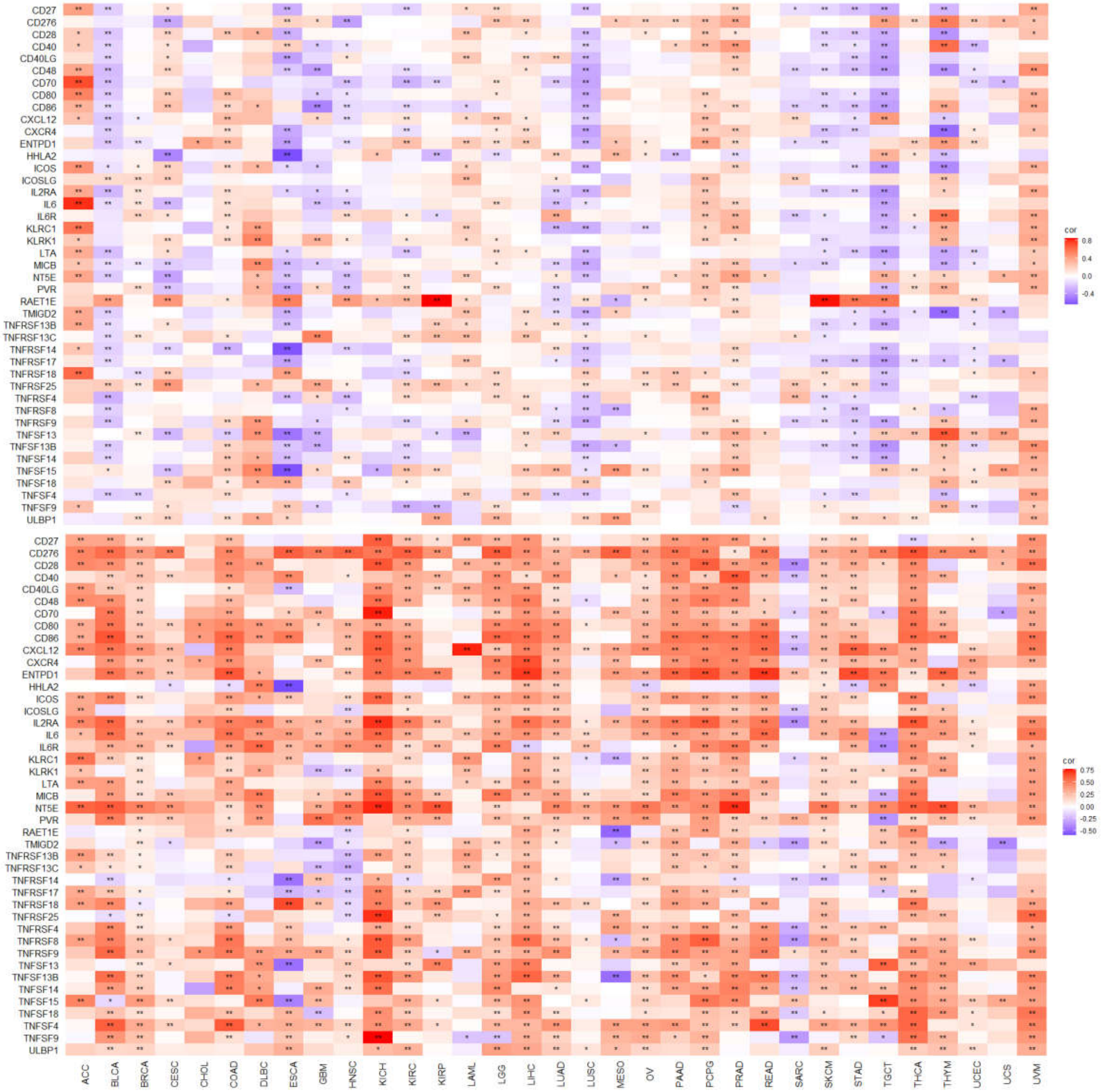
CD27
CD276
CD28
CD40
CD40LG
CD48
CD70
CD80
CD86
CXCL12
CXCR4
ENTPD1
HHLA2
ICOS
ICOSLG
IL2RA
IL6
IL6R
KLRC1
KLRK1
LTA
MICB
NT5E
PVR
RAET1E
TMIGD2
TNFRSF13B
TNFRSF13C
TNFRSF14
TNFRSF17
TNFRSF18
TNFRSF25
TNFRSF4
TNFRSF8
TNFRSF9
TNFSF13
TNFSF13B
TNFSF14
TNFSF15
TNFSF18
TNFSF4
TNFSF9
ULBP1
cor
0.8
0.4
0.0
-0.4
cor
0.75
0.50
0.25
0.00
-0.25
-0.50
ACC
BLCA
BRCA
CESC
CHOL
COAD
DLBC
ESCA
GBM
HNSC
KICH
KIRC
KIRP
LAML
LGG
LIHC
LUAD
LUSC
MESO
OV
PAAD
PCPG
PRAD
READ
SARC
SKCM
STAD
TGCT
THCA
THYM
UCEC
UCS
UVM

Supplemental Figure 11

The pan-cancer ICGs correlation analysis demonstrates the expression level correlation between immune-stimulating genes and either ZNF750 (upper panel) or TNC (lower panel) in 33 types of cancer. This analysis was conducted using the TCGA-plot R package.

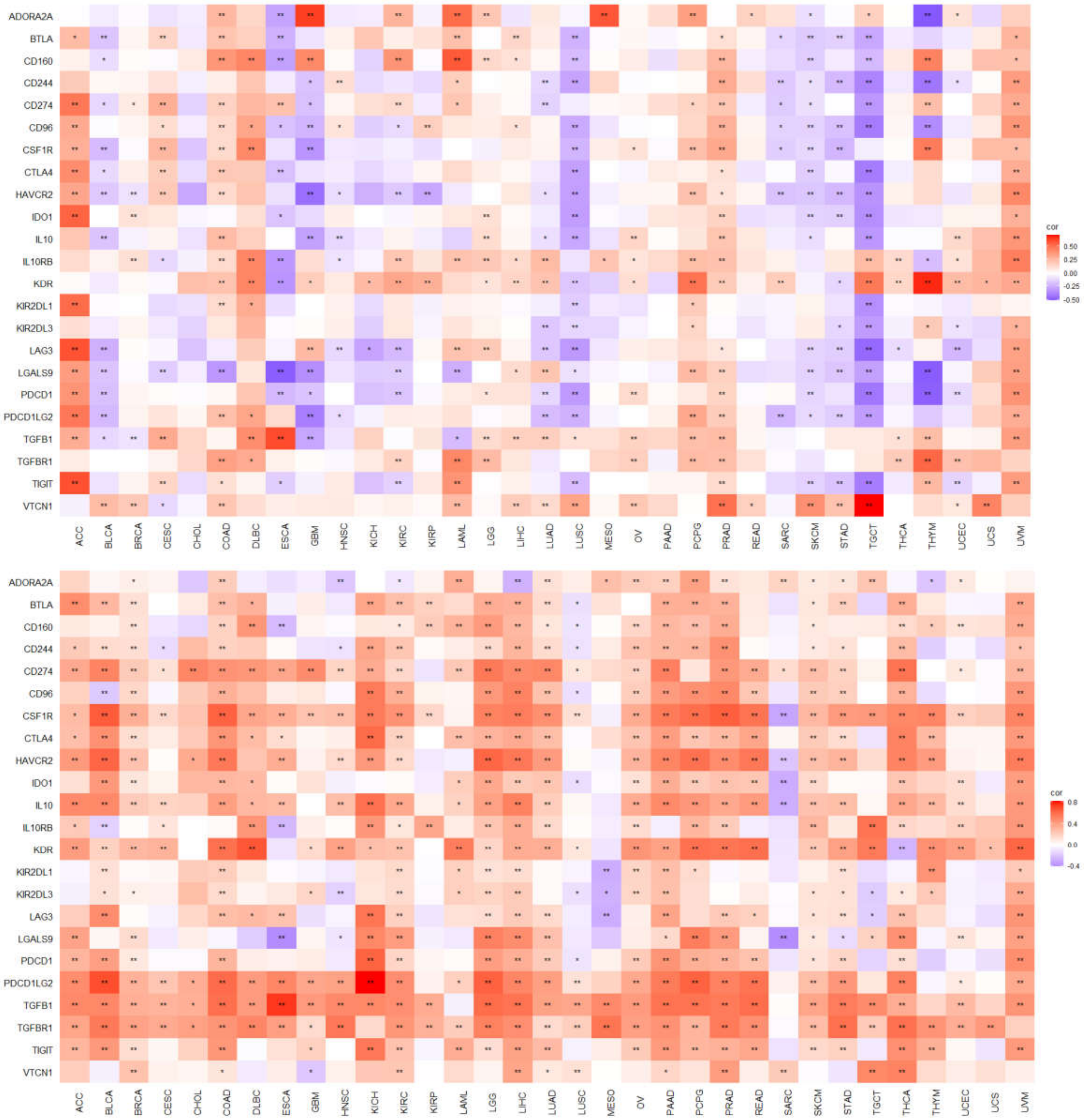
ADORA2A
BTLA
CD160
CD244
CD274
CD96
CSF1R
CTLA4
HAVCR2
IDO1
IL10
IL10RB
KDR
KIR2DL1
KIR2DL3
LAG3
LGALS9
PDCD1
PDCD1LG2
TGFB1
TGFBR1
TIGIT
VTCN1
ACC BLCA BRCA CESC CHOL COAD DLBC ESCA GBM HNSC KICH KIRC KIRP LAML LGG LIHC LUAD LUSC MESO OV PAAD PCPG PRAD READ SARC SKCM STAD TGCT THCA THYM UCEC UCS UVM
cor
0.50
0.25
0.00
-0.25
-0.50
cor
0.8
0.4
0.0
-0.4

Supplemental Figure 12

The pan-cancer ICGs correlation analysis demonstrates the expression level correlation between immune-inhibitory genes and either ZNF750 (upper panel) or TNC (lower panel) in 33 types of cancer. This analysis was conducted using the TCGA-plot R package.

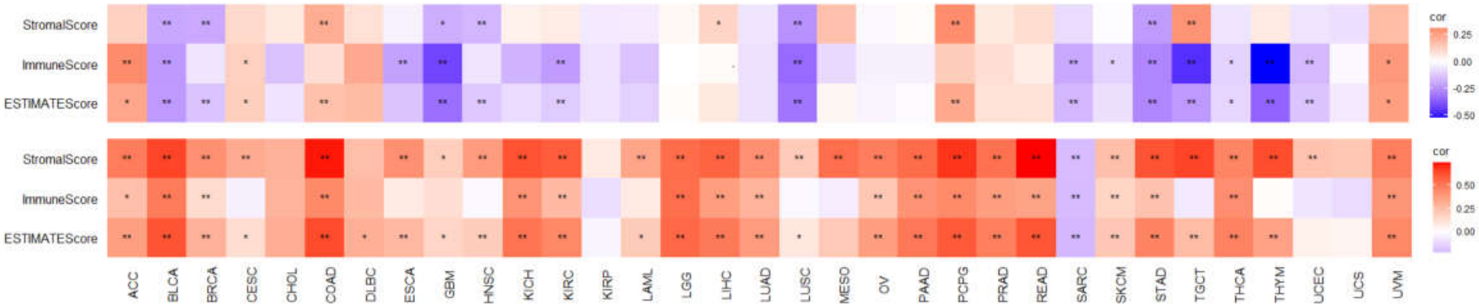

StromalScore
ImmuneScore
ESTIMATEScore
cor
0.25
0.00
-0.25
-0.50
0.50
ACC
BLCA
BRCA
CESC
CHOL
COAD
DLBC
ESCA
GBM
HNSC
KICH
KIRC
KIRP
LAML
LGG
LIHC
LUAD
LUSC
MESO
OV
PAAD
PCPG
PRAD
READ
SARC
SKCM
STAD
TGCT
THCA
THYM
UCEC
UCS
UVM

Supplemental Figure 13

The pan-cancer ICGs correlation analysis demonstrates the expression level correlation between immunity score and either ZNF750 (upper panel) or TNC (lower panel) in 33 types of cancer. This analysis was conducted using the TCGA-plot R package.

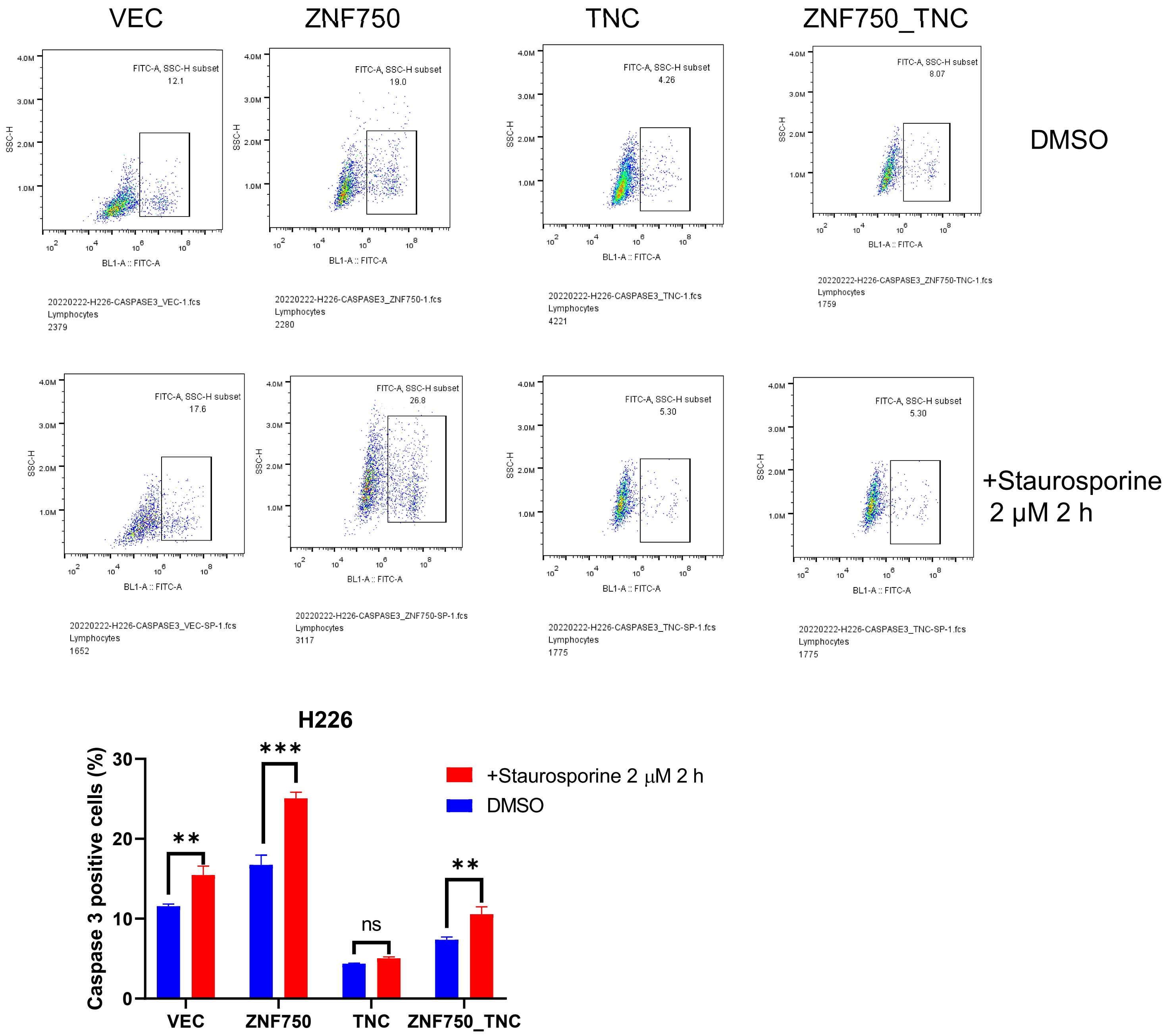
VEC
ZNF750
TNC
ZNF750_TNC
DMSO
+Staurosporine 2 μM 2 h
FITC-A, SSC-H subset
12.1
19.0
4.26
8.07
17.6
26.8
5.30
5.30
SSC-H
BL1-A :: FITC-A
20220222-H226-CASPASE3_VEC-1.fcs
Lymphocytes
2379
20220222-H226-CASPASE3_ZNF750-1.fcs
Lymphocytes
2280
20220222-H226-CASPASE3_TNC-1.fcs
Lymphocytes
4221
20220222-H226-CASPASE3_ZNF750-TNC-1.fcs
Lymphocytes
1759
20220222-H226-CASPASE3_VEC-SP-1.fcs
Lymphocytes
1652
20220222-H226-CASPASE3_ZNF750-SP-1.fcs
Lymphocytes
3117
20220222-H226-CASPASE3_TNC-SP-1.fcs
Lymphocytes
1775
20220222-H226-CASPASE3_TNC-SP-1.fcs
Lymphocytes
1775
H226
Caspase 3 positive cells (%)
+Staurosporine 2 μM 2 h
DMSO
**
***
ns
**
VEC
ZNF750
TNC
ZNF750_TNC

Supplemental Figure 14

The flow cytometry results of caspase 3 in the H226 cell line treated with or without the apoptosis inducer Staurosporine. The statistical analysis of these results is also included, multiple *t* test, ns: $P > 0.05$, *: $P < 0.05$, **: $P < 0.01$, ***: $P < 0.001$.

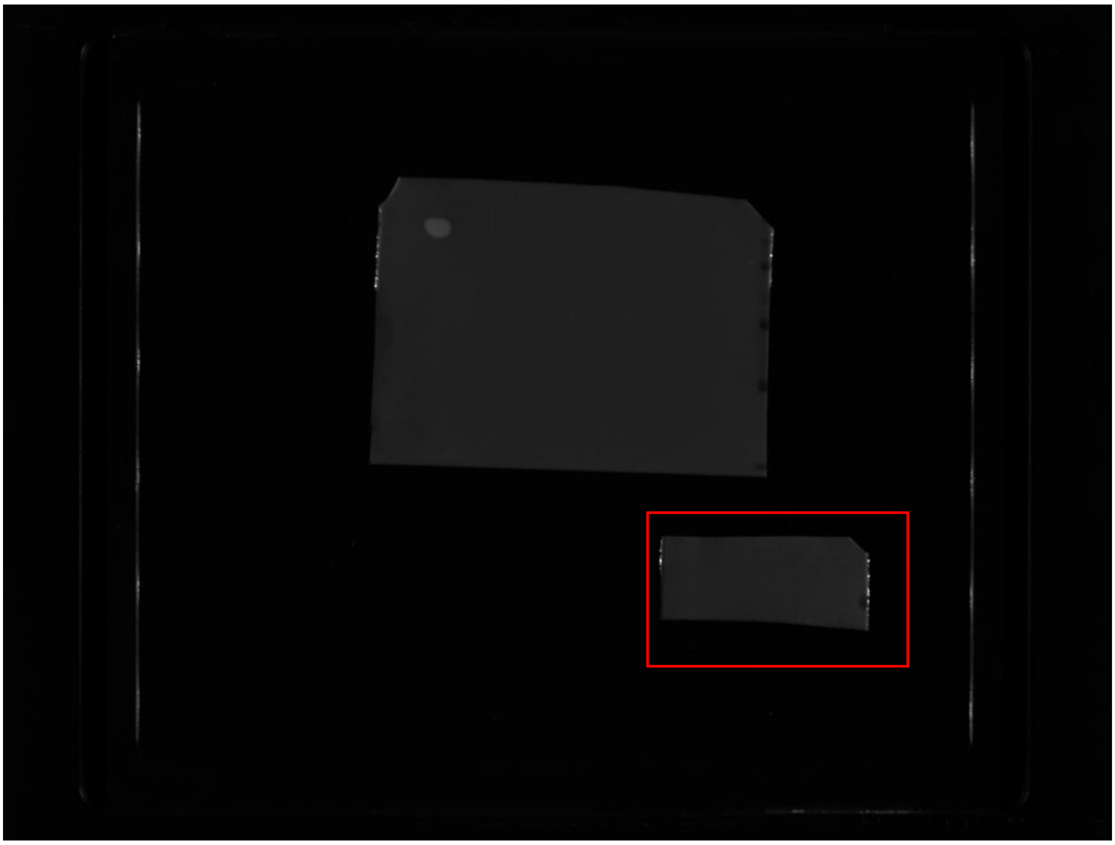

Actin

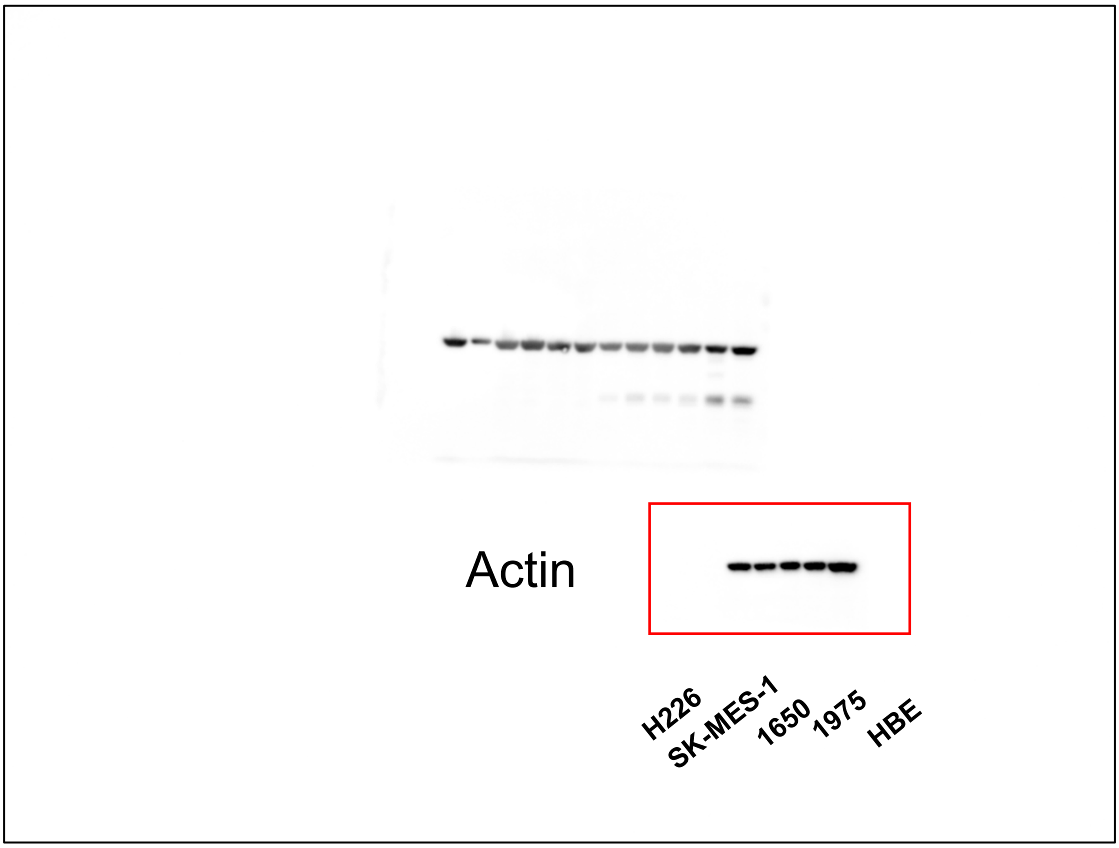

Supplemental Figure 15.

Full length blots of actin in Figure 1A.

ZNF750

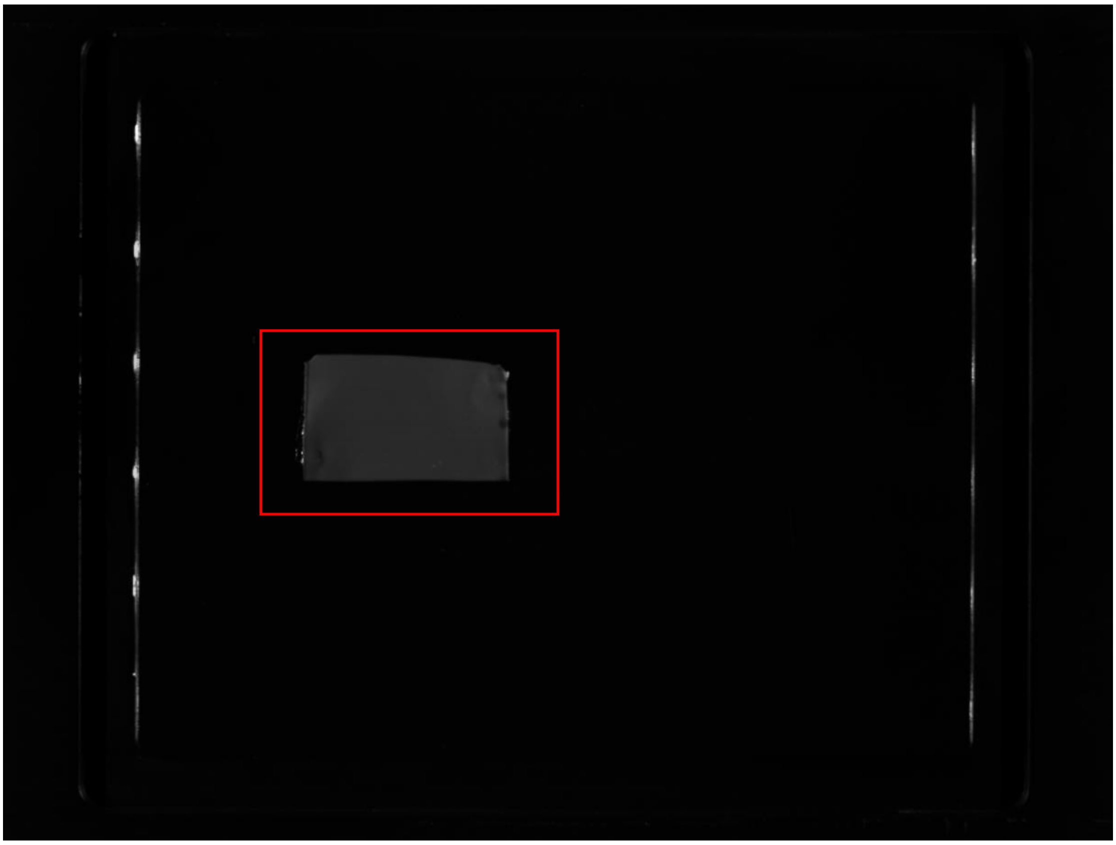

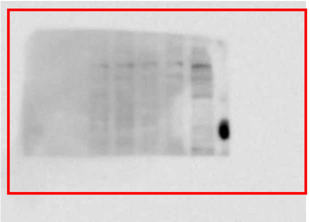

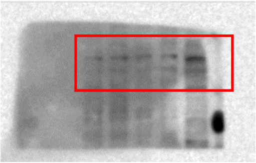

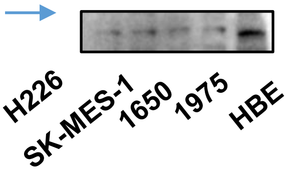

ZNF750

Supplemental Figure 16.

Full length blots of ZNF750 in Figure 1A.

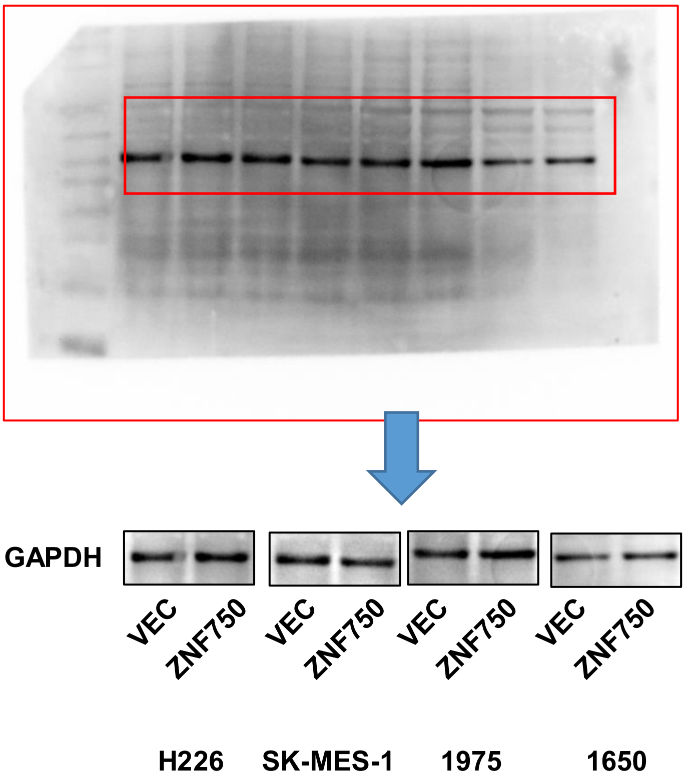
GAPDH
VEC
ZNF750
VEC
ZNF750
VEC
ZNF750
VEC
ZNF750
H226
SK-MES-1
1975
1650

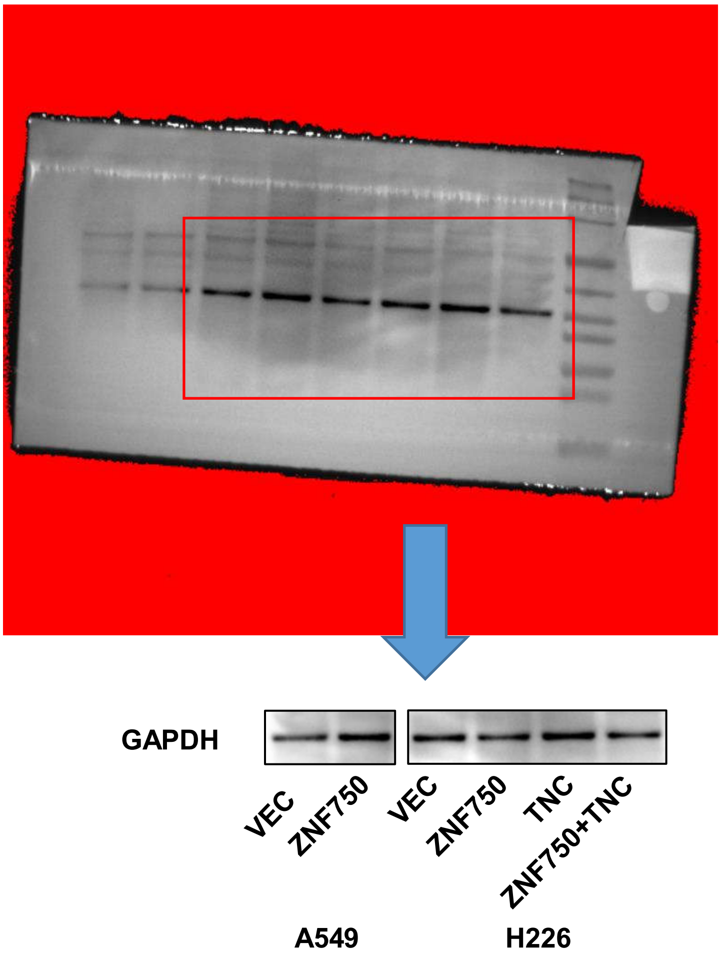
GAPDH
VEC
ZNF750
VEC
ZNF750
TNC
ZNF750+TNC
A549
H226

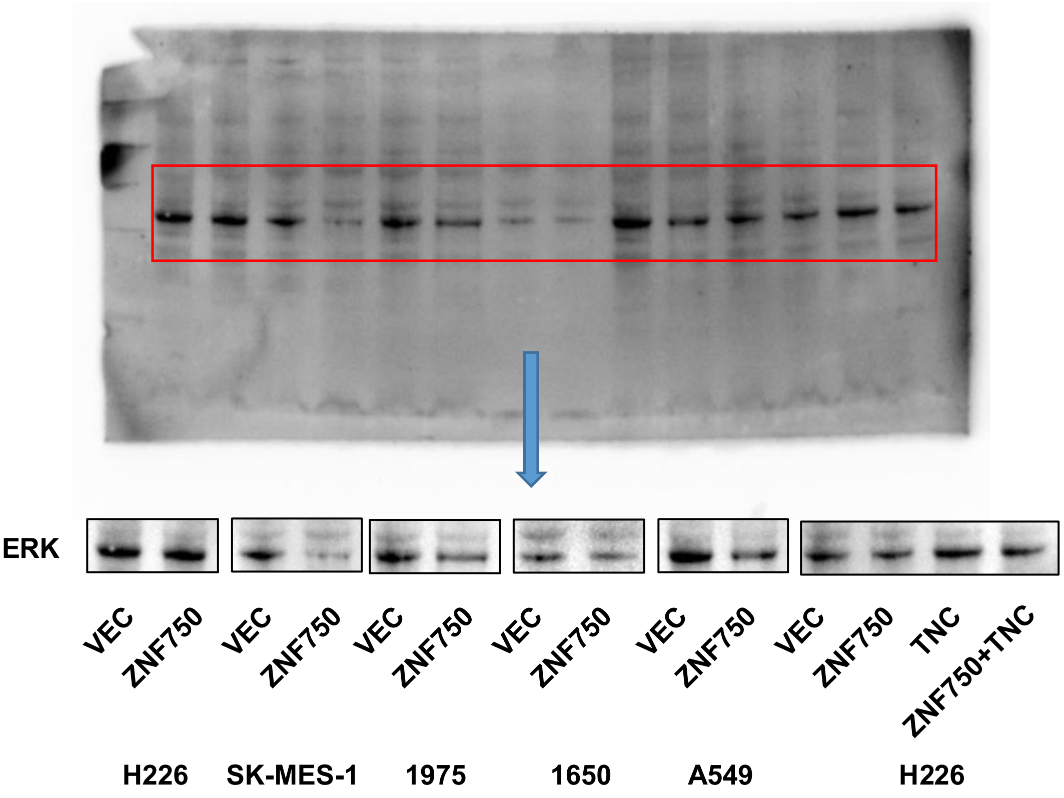
ERK
VEC
ZNF750
VEC
ZNF750
VEC
ZNF750
VEC
ZNF750
VEC
ZNF750
VEC
ZNF750
TNC
ZNF750+TNC
H226
SK-MES-1
1975
1650
A549
H226

Supplemental Figure 17.

Full length blots of GAPDH & ERK in Figure 7C.

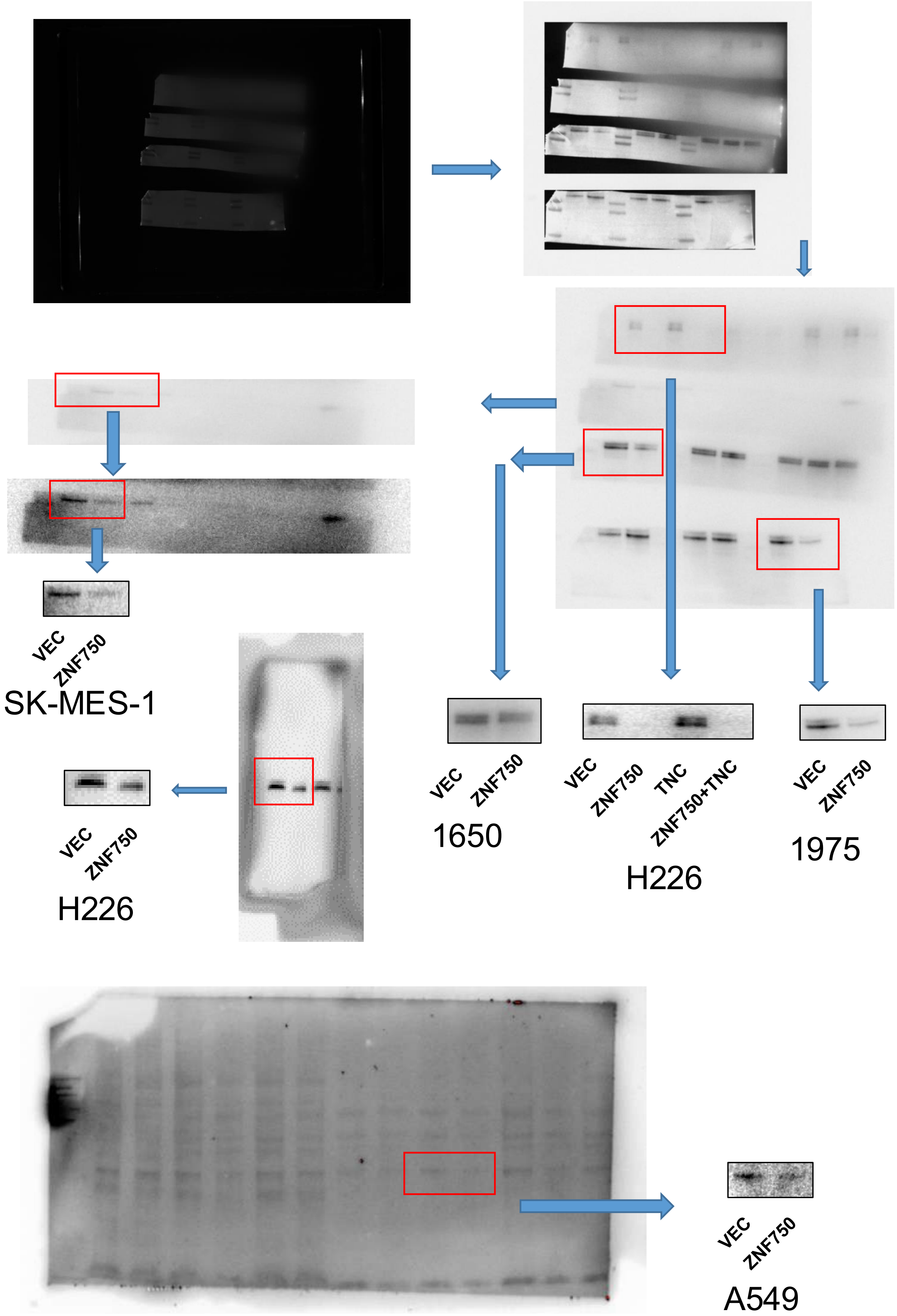
VEC
ZNF750
SK-MES-1
VEC
ZNF750
H226
VEC
ZNF750
1650
VEC
ZNF750
TNC
ZNF750+TNC
H226
VEC
ZNF750
1975
VEC
ZNF750
A549

Supplemental Figure 18.

Full length blots of pERK in Figure 7C.

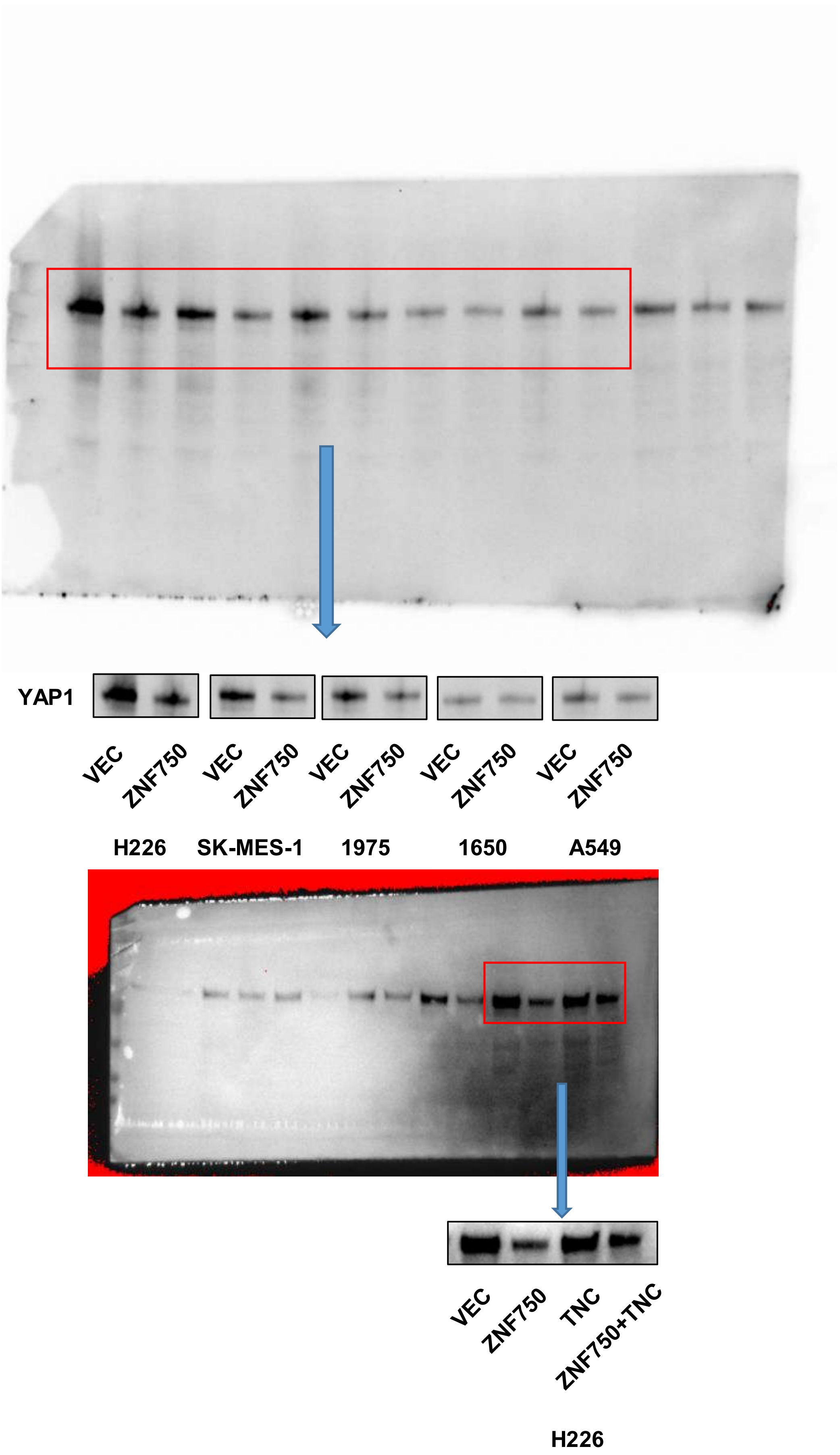

YAP1
VEC
ZNF750
VEC
ZNF750
VEC
ZNF750
VEC
ZNF750
VEC
ZNF750
H226
SK-MES-1
1975
1650
A549
VEC
ZNF750
TNC
ZNF750+TNC
H226

Supplemental Figure 19.

Full length blots of YAP1 in Figure 7C.

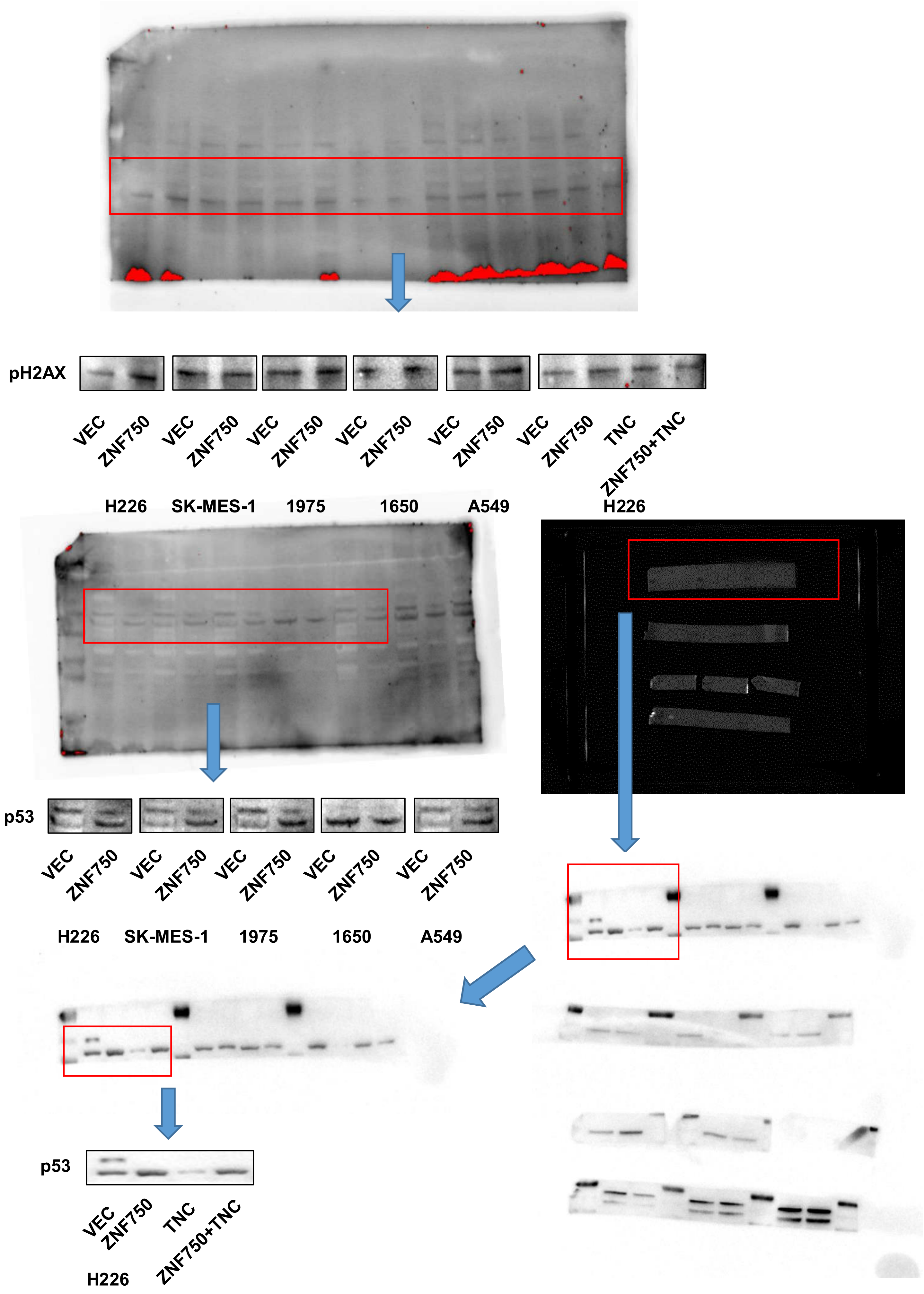
pH2AX
VEC
ZNF750
VEC
ZNF750
VEC
ZNF750
VEC
ZNF750
VEC
ZNF750
VEC
ZNF750
TNC
ZNF750+TNC
H226
SK-MES-1
1975
1650
A549
H226
p53
VEC
ZNF750
VEC
ZNF750
VEC
ZNF750
VEC
ZNF750
VEC
ZNF750
H226
SK-MES-1
1975
1650
A549
p53
VEC
ZNF750
TNC
ZNF750+TNC
H226

Supplemental Figure 20.

Full length blots of pH2AX & p53 in Figure 7C.

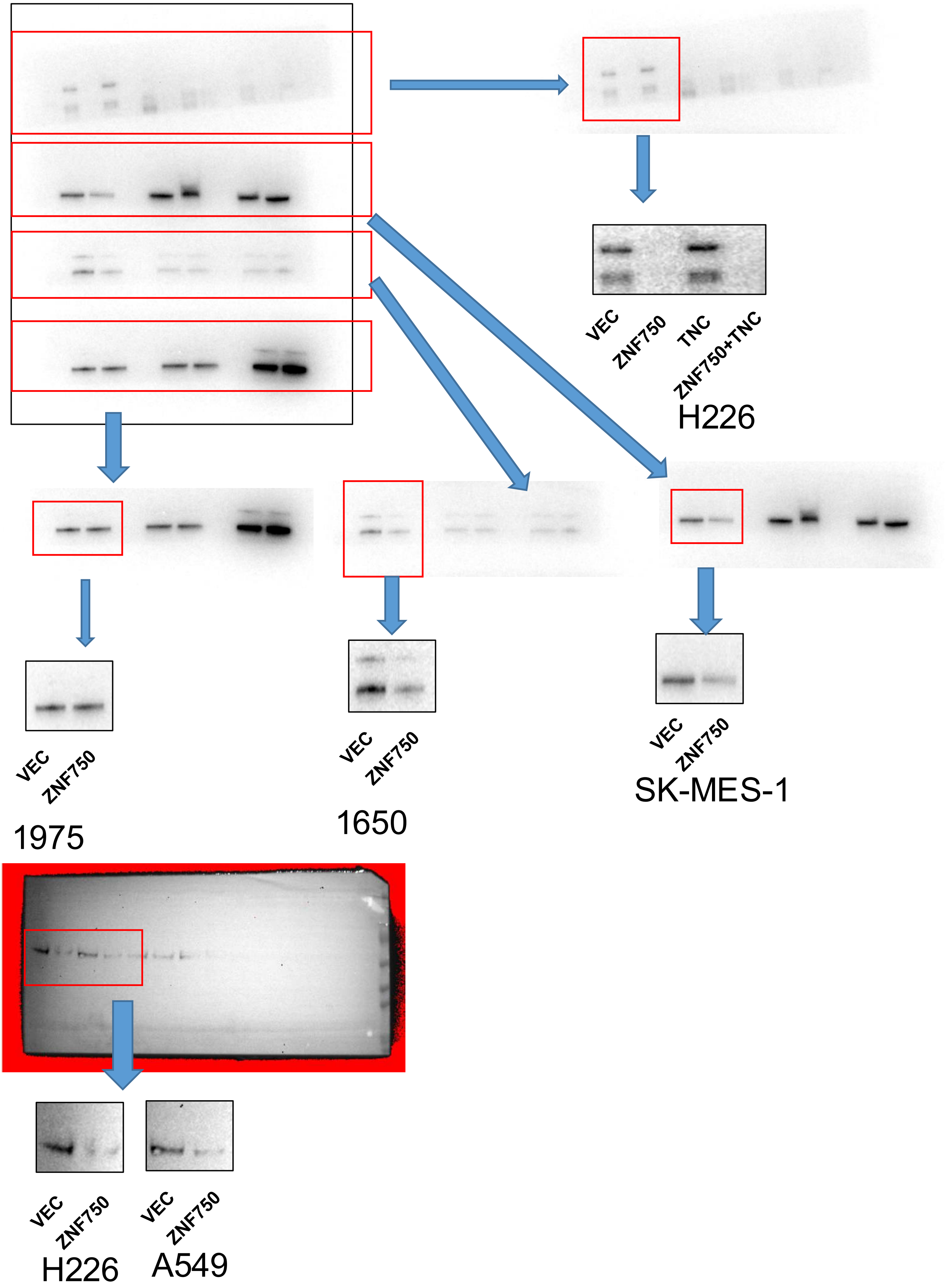
VEC
ZNF750
TNC
ZNF750+TNC
H226
VEC
ZNF750
1975
VEC
ZNF750
1650
VEC
ZNF750
SK-MES-1
VEC
ZNF750
H226
VEC
ZNF750
A549

Supplemental Figure 21.

Full length blots of YAP/TAZ in Figure 7C.

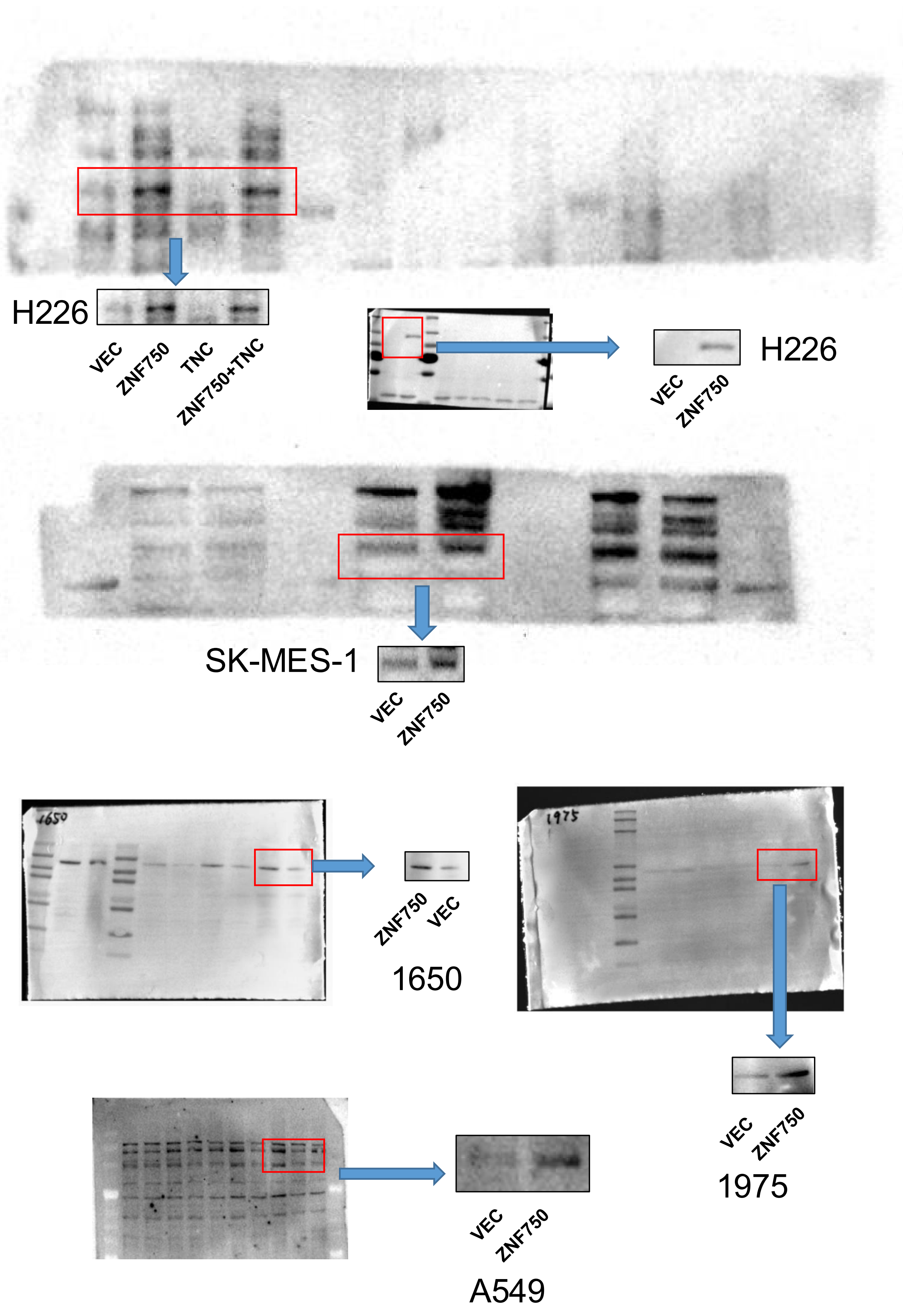
H226
VEC
ZNF750
TNC
ZNF750+TNC
H226
VEC
ZNF750
SK-MES-1
VEC
ZNF750
ZNF750
VEC
1650
VEC
ZNF750
1975
VEC
ZNF750
A549

Supplemental Figure 22.

Full length blots of ZNF750 in Figure 7C.
