# Supplementary material for: Tenascin C as a novel zinc finger protein 750 target regulating the immunogenicity via DNA damage in lung squamous cell carcinoma
Source: BMC Cancer. 2024 May 6;24:561. doi: 10.1186/s12885-024-12285-8 (PMC11071264; doi:10.1186/s12885-024-12285-8)
Supplement: Supplementary file 2 — Supplementary Material 2. [file 12885_2024_12285_MOESM2_ESM.docx]

*Tenascin C as a Novel Zinc Finger Protein 750 Target Regulating the Immunogenicity via DNA Damage in Lung Squamous Cell Carcinoma*

*Supplementary Materials and Methods*

# Plasmids transfection and lentiviral infection

The plasmids used in the study are listed in Supplementary Table1. Plasmids transfections were carried out with lipofetamine 3000 (Invitrogen, USA) according to the manufacturer’s protocols. Lentiviral packaging was applied by 293T cells transfections of two packaging plasmids and the lentiviral vector encoding target sequence. 48 hours after Lenti-viral plasmids transfection, the supernatant of 293T cells was filtered by 0.45 μm Millex® -HP filter (#SLHPR33RB, Merck Millipore Ltd., IRELAND) and then added to target cells at 1:1 supernatant: medium volume ratio.

**Immunoblot analysis**

Samples containing equal amounts of protein (30 μg) were separated on a 7.5% or 10% sodium dodecylsulphate polyacrylamide gel electrophoresis (SDS-PAGE) and were transferred electrophoretically onto Immobilon®-P membrane with 0.45μm pore size (#IPVH00010, Merck Millipore Ltd.). Membranes were probed with the appropriate polyclonal or monoclonal primary antibody, and then incubated with horseradish peroxidase-conjugated anti-rabbit, anti-mouse or anti-goat immunoglobulin G. Immunoreactive proteins were visualized by ECL Western Blotting Substrate (#32106, Pierce, Rockford, IL, USA). Images of the IBs were acquired on an Azure (Azure biosystems, CA, USA). The antibodies used for western blotting were listed in Supplementary Table 1. Some of the blots were trimmed prior to hybridization with antibodies, so we added the merged picture showing the overlap between bright field view and the chemical luminescence view in **Supplemental Figure 15-22**.

# Immunefluorescence

Cells for Immunefluorescence were seeded in wells of Millicell EZ slide (#PEZGS0816, Merck Millipore Ltd.). After treated with described drug, the cells were washed with ice-cold PBS and fixed with 4% paraformaldehyde in PBS for 10 minutes at room temperature. After washing three times with PBS, cells were permeabilized with 0.1% TritonX100 in PBS. Then nonspecific binding was blocked with 3% of bovine serum albumin (BSA, #A8020, Solarbio) in PBS for 1 hour at room temperature. Fixed cells were incubated with the primary antibody overnight at 4 °C, washed three times with 0.01% TritonX100 in PBS and then incubation with the secondary antibody for 2 hours at room temperature. Sections were mounted in Mounting Reagent (#ZLI-9557, ZSGB-BIO,) with 24*40 mm cover glass for imaging. Antibodies used were listed in Supplementary Table 1. Images were obtained using laser scanning confocal microscopy (TCS-SP8, Leica, Germany) with LASX software.

# Scanning electron microscopy

Seed cells on a sterile cover glass in a petri dish, followed by adding electron microscopy fixative into petri dish. Post-fix, dehydrate, and drying. Specimens are attached to metallic stubs using carbon stickers and sputter-coated with gold for 30 s. Then, the ultrastructure of the corneal endothelium was observed by scanning electron microscope (SEM, JSM6390LV, JEOL, Japan) after dehydration, drying, and gold plating.

# Transwell assay

A transwell filter with 8 μm pores (#TCS020024, BIOFIL, China) were placed into 24-well plates. The cell density was adjusted to 1×10^5^ cells/mL, and 200μL of cell suspension in serum-free medium was added to the upper well. About 700μL of medium containing 10% FBS was added to the lower chamber and incubated at 37°C for 48 h. The cells on the lower surface of the membrane were washed twice with PBS, and the remaining cells on the upper of the membrane were wiped off with a wet cotton swab. The cells were immobilized with methanol for 30 min, stained with 0.5% crystal violet for 15 min, rinsed with running tap water for 1 min. Air dry and photographed under a microscope and counted using Image J.

# Colony formation

For colony formation assay, cells were plated in the 6 cm dish at a density of 1000 cells/mL for 14-21 days to form spheres. Colonies were fixed with 4% paraformaldehyde for 20 min and then stained with 1% crystal violet for 15 min, then rinsed with tap water and air dried. Colonies were counted and surviving fraction was calculated as the mean number of colonies.

# In vitro cytotoxicity study

The cell viability was investigated using the standard CCK8 assay according to the manufacture’s instruction (MA0218, Meilunbio, China). In brief, the cells were seeded at a density of 5 × 10^3^ cells per well in the 96 well plates, and cultured for 24, 48, 72 hours, respectively. The CCK8 reagent (10 µL) was added to each well and incubated for another 4 h. The absorbance at 450 nm was measured by a microplate reader (Bio-rad, USA). The cell viability was calculated by comparing the ratio of OD values.

# Cell cycle and apoptosis analysis

Cell cycle analysis and measurement of the percentage of apoptotic cells were performed by flow cytometry. Cells were seeded at 1×10 ^6^ cells per well in 6-well plates. After 12 hours growth, the medium was replaced with maintenance medium containing corresponding drugs or directly applied following staining processes. After treatment, floating cells in the medium were combined with attached cells collected by trypsinization. Cell cycle distribution analysis was performed as follows, cells were fixed and permeabilized using 70% ethanol in PBS, prior to staining with PI Staining Buffer (#C0080, Solarbio, China). Levels of apoptosis were analyzed using the FITC Annexin V- Apoptosis Detection Kit I(#556547, BD Pharmingen) or APC Annexin V Apoptosis Detection Kit with 7-AAD(#640930, Biolegend) following the instructions of the manufactures. The cells were analyzed by flow cytometry within 1 h of staining. Analysis was performed using FlowJo software (FlowJo, LLC). A complete list of antibodies and other reagents utilized is available in Supplementary Table 1. In Supplemental Figure 14， the medium was replaced with maintenance medium containing 2 μM Staurosporine (#HY-15141, Medchemexpress) or DMSO for H226 cells. The cells were incubated for 3 hours and staining with FITC active caspase-3 apoptosis kit (#550480, BD Biosciences).

# ChIP-seq data analysis

FASTQ files were generated with Illumina’s Bcl2Fastq tool (v2.20). Quality control and adapter removal was performed using FASTQ（v 0.21.0）^[1]^.All quality paired-end reads were mapped to hg19 reference genome using hisat2 (v 2.2.1)^[2]^ and SAMtools (v 1.15.1)^[3]^[3], deduplicated using Picard (v 2.23.8) (http://broadinstitute.github.io/picard/) MarkDuplicates with default settings. Peaks were calculated using MACS2 (v 2.2.7.1) ^[4]^, callpeak using setting --SPMR -f BAM -g hs -B --qvalue-cutoff 0.05. Peaks annotation were performed by ChIPSeeker R package ^[5]^.Genomic tracks were visualized using Integrated Genome Viewer (IGV) (v 2.3) ^[6]^ . Heatmaps and histogram were depicted using computeMatrix and plotHeatmap in deepTools (v 3.5.0) ^[7]^.

# RNA-seq quality control and sequencing

RNA was extracted by Trizol method, Total RNA quality is determined by estimating the A260/ A280 and A260/A230 ratios by nanodrop. RNA integrity is determined by running an Qiaxcel Bioanalyzer gel, which measures the ratio of the ribosomal peaks.The preparation and sequencing of the RNA sequencing library were completed by Amogene(Xiamen, China) and Novogene(Tianjin, China) respectively. The steps are briefly described as follows: Paired-end sequencing was performed using Illumina NovaSeq 6000 on the cDNA library constructed with NEBNext Poly(A) mRNA Magnetic Isolation Module(#E7490, NEB) and NEBNext Ultra II Directional RNA Library Prep Kit for Illumina (#E7760, NEB).

# RNA-seq data analysis

Quality control and adapter removal was performed using fastp.The resultant high-quality reads were mapped to the hg19 reference genome using hisat2 and samtools. Assembly was completed using StringTie (2.1.5)^[8]^ with parameter -e -B --rf. Differential gene expression analysis was performed using DESeq2 (1.30.1)^[9]^. Pathway and Ontology enrichment analysis were performed using Metascape for differentially expressed genes with a significance cutoff of *P* value <0.05 and Log2FC absolute value >1. GSEA analysis was applied with pre-ranked gene list. Soft clustering of gene expression data in different groups was conducted using Mfuzz (v2.50.0)^[10]^ R package, and the cluster of gene sets enrichment analysis were performed using metascape.

# scRNA-seq Data source and analysis

The scRNA-seq dataset GSE148071 of LUAD and LUSC were downloaded from the GEO (https://www.ncbi.nlm.nih.gov/) database, containing scRNA-seq of tumor tissues from 20 LUAD and 22 LUSC patients, the patients information and sequencing statistics were shown in Supplementary Table 2^[11]^.The samples were integrate using anchors method in the R package "Seurat" and core cells were obtained by filtering scRNA-seq^[12]^. Ineligible cells include genes that can only be detected in 3 or fewer cells and low-quality cells with less than 200 genes detected will be excluded from subsequent analysis. Gene expression of core cells was normalized using a linear regression model, and then the top 2000 genes with highly variable characteristics were screened by ANOVA. The first 20 principle components and resolution 1.0 were used with FindClusters function to generate 33 cell clusters. To assign one of the 13 major cell types to each cluster, we scored each cluster by the normalized expressions of the following canonical markers were shown in Supplementary Table 3. The highest scored cell type was assigned to each cluster. Cancer cell clusters were negative for normal lung epithelial markers and positive for EPCAM. The clusters assigned to the same cell type were lumped together for the following analysis. The final results were manually examined to ensure the correctness of the results and visualized by Uniform Manifold Approximation and Projection (UMAP)^[13]^. The 13 major cell types were chosen by initial exploratory inspection of the differentially expressed genes (DEGs) of each cluster combined with literature study. The DEGs were generated by Seurat FindMarkers function. After identifying cell types in RA synovium, cell-cell communication was analyzed by implementing the CellChat (V.1.1.3) pipeline ^[14]^. A new CellChat object was created from the merged Seurat object. The paracrine/autocrine signaling interaction dataset of CellChatDB was set as referencing database. Next, the communication probability was computed using a truncated mean of 20% (function computeCommunProb, type = "truncatedMean", trim = 0.2). After that, the cell-cell communication was inferred and the cell-cell communication network was aggregated with default parameters. The number of interactions was visualized to show the aggregated cell-cell communication network and signaling sent from each cell cluster.

# RT-qPCR

RNA was extracted by TRIZOL and was reverse-transcripted using HiFiScript gDNA Removal RT Master Mix(#CW2020, CWBIO) according to the manufacture’s instruction. The primers used for quantitative RT-PCR analyses are described in Supplementary Table 1.

# Dual luciferase promoter assay

ZNF750 and VEC cells were plated in 12-well plates the day before transfection. After 16 hours co-transfection with empty vector *pGL4.27[luc2P/minP/Hygro]* (#E845A, Promega) or gene promoter vector *pGL4.27-TNC-promoter* (421bp) and *pGL4.75[hRluc/CMV]* #E6931, Promega) as an internal control, the dual luciferase reporter assay kit (#E1910, Promega,) was used to determine gene promoter activity according to the protocols provided by the manufacturer. The data was represented as the relative luciferase activity by the means of ratio of firefly luciferase to Renilla luciferase activity by six replicates.

# Hematoxylin and eosin (HE) staining and immunohistochemistry (IHC)

We obtained 90 cases of formalin-fixed paraffin-embedded (FFPE) LUSC tissue samples from Shanghai Outdo biotech CO.,LTD. These samples were from LUSC patients who had undergone surgery from July 2004 to May 2008. The FFPE sections were deparaffinized, followed by hematoxylin staining, eosin staining, dehydration, and sealing the slide with neutral gum for examination under a microscope. The FFPE sections were deparaffinized in xylene, rehydrated through a graded series of ethanol and phosphate-buffered saline (PBS), and incubated in 3% H2O2 for 10 min to block endogenous peroxidase activity. After antigen retrieval by microwave heating (95 °C for 20 min) in 10 mM Tris/1 mM EDTA buffer, the sections were incubated overnight at 4 °C with a primary monoclonal antibody. The sections were then incubated with EnVision (Maxim) for 30 min at room temperature, and color was developed using 3,3′-diaminobenzidine tetrachloride (Maxim) as the chromogen. The slides were subsequently counterstained with hematoxylin. Appropriate positive and negative controls were used in each experiment. The antibodies used are listed in Supplementary Table 1.

# Pan-cancer immune checkpoint genes correlation analysis

The pan-cancer ICGs correlation analysis were applied using TCGA-plot R package^[15]^ the expression level correlation between immune checkpoint genes and either ZNF750 (upper panel) or TNC (lower panel) in 33 types of cancer.

# Statistical analysis

All in vitro experiments were applied in 3 replicants except for ChIP-seq(no replicants) and Dual luciferase promoter assay(6 replicants). Data was analyzed using R packages unless otherwise indicated. The specific statistical analysis methods were all specified in the legends otherwise in the related experimental methods.

# Reference of Supplementary Materials and Methods.

1. Chen S, Zhou Y, Chen Y, Gu J. fastp: an ultra-fast all-in-one FASTQ preprocessor. **Bioinformatics** **2018**, 34(17)**:** i884-i890.

2. Kim D, Langmead B, Salzberg SL. HISAT: a fast spliced aligner with low memory requirements. **Nat Methods** **2015**, 12(4)**:** 357-360.

3. Li H, Handsaker B, Wysoker A, Fennell T, Ruan J, Homer N*, et al.* The Sequence Alignment/Map format and SAMtools. **Bioinformatics** **2009**, 25(16)**:** 2078-2079.

4. Liu T. Use model-based Analysis of ChIP-Seq (MACS) to analyze short reads generated by sequencing protein-DNA interactions in embryonic stem cells. **Methods Mol Biol** **2014**, 1150**:** 81-95.

5. Yu G, Wang LG, He QY. ChIPseeker: an R/Bioconductor package for ChIP peak annotation, comparison and visualization. **Bioinformatics** **2015**, 31(14)**:** 2382-2383.

6. Robinson JT, Thorvaldsdóttir H, Winckler W, Guttman M, Lander ES, Getz G*, et al.* Integrative genomics viewer. **Nat Biotechnol** **2011**, 29(1)**:** 24-26.

7. Ramírez F, Dündar F, Diehl S, Grüning BA, Manke T. deepTools: a flexible platform for exploring deep-sequencing data. **Nucleic Acids Res** **2014**, 42(Web Server issue)**:** W187-191.

8. Pertea M, Pertea GM, Antonescu CM, Chang TC, Mendell JT, Salzberg SL. StringTie enables improved reconstruction of a transcriptome from RNA-seq reads. **Nat Biotechnol** **2015**, 33(3)**:** 290-295.

9. Love MI, Huber W, Anders S. Moderated estimation of fold change and dispersion for RNA-seq data with DESeq2. **Genome Biol** **2014**, 15(12)**:** 550.

10. Kumar L, M EF. Mfuzz: a software package for soft clustering of microarray data. **Bioinformation** **2007**, 2(1)**:** 5-7.

11. Wu F, Fan J, He Y, Xiong A, Yu J, Li Y*, et al.* Single-cell profiling of tumor heterogeneity and the microenvironment in advanced non-small cell lung cancer. **Nat Commun** **2021**, 12(1)**:** 2540.

12. Gribov A, Sill M, Lück S, Rücker F, Döhner K, Bullinger L*, et al.* SEURAT: visual analytics for the integrated analysis of microarray data. **BMC Med Genomics** **2010**, 3**:** 21.

13. Becht E, McInnes L, Healy J, Dutertre CA, Kwok IWH, Ng LG*, et al.* Dimensionality reduction for visualizing single-cell data using UMAP. **Nat Biotechnol** **2018**.

14. Jin S, Guerrero-Juarez CF, Zhang L, Chang I, Ramos R, Kuan CH*, et al.* Inference and analysis of cell-cell communication using CellChat. **Nat Commun** **2021**, 12(1)**:** 1088.

15. Liao C, Wang X. TCGAplot: an R package for integrative pan-cancer analysis and visualization of TCGA multi-omics data. **BMC Bioinformatics** **2023**, 24(1)**:** 483.
